# Wetland productivity determines trade‐off between biodiversity support and greenhouse gas production

**DOI:** 10.1002/ece3.10619

**Published:** 2023-10-20

**Authors:** David Åhlén, Mike Peacock, Yngve Brodin, Peter A. Hambäck

**Affiliations:** ^1^ Department of Ecology, Environment and Plant Sciences Stockholm University Stockholm Sweden; ^2^ Department of Aquatic Sciences and Assessment Swedish University of Agricultural Sciences Uppsala Sweden; ^3^ Department of Geography and Planning, School of Environmental Sciences University of Liverpool Liverpool UK; ^4^ Department of Zoology The Swedish Museum of Natural History Stockholm Sweden

**Keywords:** Chironomidae, greenhouse gasses, nutrient stoichiometry, primary production, trade‐offs, wetlands

## Abstract

Establishing wetlands for nutrient capture and biodiversity support may introduce trade‐offs between environmentally beneficial functions and detrimental greenhouse gas emissions. Investigating the interaction of nutrient capture, primary production, greenhouse gas production and biodiversity support is imperative to understanding the overall function of wetlands and determining possible beneficial synergistic effects and trade‐offs. Here, we present temporally replicated data from 17 wetlands in hemi‐boreal Sweden. We explored the relationship between nutrient load, primary producing algae, production of methane and nitrous oxide, and emergence rates of chironomids to determine what factors affected each and how they related to each other. Chironomid emergence rates correlated positively with methane production and negatively with nitrous oxide production, where water temperature was the main driving factor. Increasing nutrient loads reduced methanogenesis through elevated nitrogen concentrations, while simultaneously enhancing nitrous oxide production. Nutrient loads only indirectly increased chironomid emergence rates through increased chlorophyll‐*a* concentration, via increased phosphorus concentrations, with certain taxa and food preference functional groups benefitting from increased chlorophyll‐*a* concentrations. However, water temperature seemed to be the main driving factor for chironomid emergence rates, community composition and diversity, as well as for greenhouse gas production. These findings increase our understanding of the governing relationships between biodiversity support and greenhouse gas production, and should inform future management when constructing wetlands.

## Graphical abstract

We investigated potential synergies or trade‐offs between chironomid community metrics and greenhouse gas production in wetlands based on wetland productivity. We found clear trade‐offs between chironomids and methane production, with the opposite relationship to nitrous oxide. The main drivers for this seemed to be seasonal variation and nutrient stoichiometric effects on productivity and greenhouse gas production.

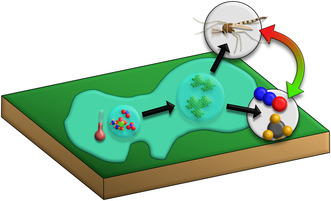

## 1 INTRODUCTION

As wetland creation has ramped up during the last four decades in response to nutrient runoff and biodiversity loss, substantial gains have been seen in reduced algal blooms in previously heavily affected areas downstream and increased abundances of many wetland species (e.g. Hsu et al., 2011; Kačergytė et al., 2021; Strand & Weisner, 2013; Thiere et al., 2009). However, wetland construction and re‐establishment come with the cost of also supporting the natural biogeochemical anaerobic and aerobic processes that are required for the production of greenhouse gases (GHGs) (Badiou et al., 2019; Lyu et al., 2018; Reddy et al., 1989). Anaerobic decomposition of organic carbon, for example through acetate splitting and CO_2_ reduction, and through aerobic and anaerobic decomposition of compound nitrogens (e.g. ammonium: ${\mathrm{NH}}_{4}^{+}$, nitrite: ${\mathrm{NO}}_{2}^{-}$ and nitrate: ${\mathrm{NO}}_{3}^{-}$), are the main sources of the powerful GHGs methane (CH_4_) and nitrous oxide (N_2_O), which are respectively 28 and 273 more warming than carbon dioxide (Forster et al., 2021; Hu et al., 2015; Lyu et al., 2018; Prosser et al., 2020; Reddy et al., 1989). Since both organic detritus and compound nitrogen are abundant in wetlands, GHG production is inevitable, and small ponds and wetlands unequivocally play important roles in global GHG budgets (Malerba et al., 2022; Peacock et al., 2021; Rosentreter et al., 2021).

Both GHG production and biodiversity are frequently regulated by nutrient load, primarily by phosphorus (P) and nitrogen (N), which affects primary production in wetlands and thereby fuels the production of detritus that drives methanogenesis and feeds aquatic organisms (Beaulieu et al., 2019; Wazbinski & Quinlan, 2013). However, system productivity may also drive GHG production and biodiversity rather than aquatic primary production alone, especially in eutrophic systems where nutrient limitation may not be the ultimate driver (Bortolotti et al., 2019). As created wetlands are often shallow with periodic‐to‐permanent inundation and high summer temperatures, their productivity often benefits semi‐aquatic organisms such as chironomid midges (Diptera: Chironomidae) (Jo et al., 2020; Leeper & Taylor, 1998; Stagliano et al., 1998), whose larvae feed on live and dead organic matter in the water but where adults are free‐flying and are then consumed in large masses by terrestrial predators (e.g. Almenar et al., 2008). The family encompasses multiple functional food preference groups (e.g. Antczak‐Orlewska et al., 2021; Armitage et al., 1995) and, as such, has inherent functional variation for divergent responses from nutrient load and primary production. Adult chironomids emerge from the water surface in great numbers during spring, summer and early autumn in northern temperate regions, being one of the most abundant insect families found around lentic wetlands (e.g. Persson Vinnersten et al., 2010, 2014). As this group is so numerous and multifaceted, it often serves as an indicator of water quality and primary production (Nicacio & Juen, 2015). In addition, emerging chironomids could form an important link between aquatic nutrient availability, aquatic primary production and prey abundance in the terrestrial food web. Several terrestrial predators such as spiders, predatory beetles, bats and various insectivorous birds, are known to feed heavily on emerging chironomid midges (de Jong & Ahlén, 1991; Lewis‐Phillips et al., 2020; Sanchez‐Ruiz et al., 2018).

Considering these processes, one may ask if there are synergistic links in these systems between the emergence of chironomids, which serve as a basis for an aquatic‐terrestrial energy flux, and a simultaneous low production of GHGs, or if these processes are instead governed by mutual trade‐offs. As wetlands supply a multitude of positive effects, but with the trade‐off of GHG emission, it is imperative to understand these interactions to get a better understanding of the net effects of the creation and management of wetlands.

In this study, we investigated how aquatic nutrient levels, temperature and phenology affected primary production, and how that relationship in turn affected CH_4_ and N_2_O concentrations, chironomid emergence rate, diversity and taxonomic and functional community complexity. We also explored if any synergistic or trade‐off relationships between chironomid emergence rates, diversity and community compositions, and GHG concentrations in these systems could be recognized. We hypothesized that wetland productivity likely drives both GHG concentrations and chironomid community metrics, and that there will be trade‐offs between factors increasing chironomid emergence rates and those increasing GHG concentrations.

## 2 METHODS

**FIGURE 1:**
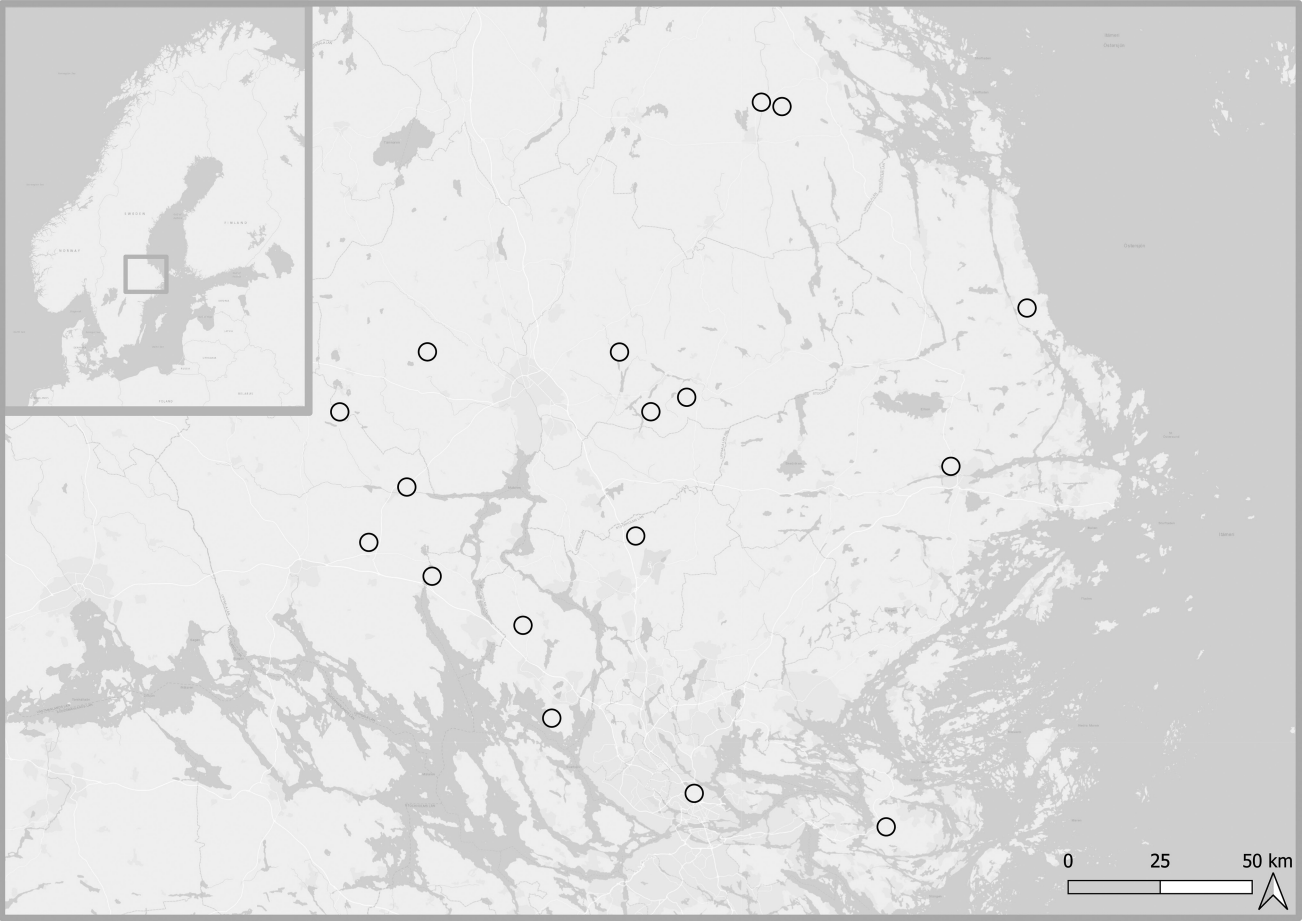
Seventeen sampling locations for Uppland, Sweden.

Data were collected from 17 lentic wetlands situated in agricultural and managed forest landscapes north of Stockholm, Sweden, and spread across a land area covering ~5600 km^2^, 59–60°N, 17–19°E (Figure 1). The region is hemi‐boreal, with a mean annual temperature of 6.5°C and annual precipitation of 576 mm. Sampling was repeated five times from May to October 2021, on an approximately monthly basis. We selected wetlands with less than 50% forest cover in a 50‐m radius and in cultivated landscapes. Included wetlands varied in morphology and management strategies and included both constructed, restored and natural wetlands (see Table S1 and Figures [Link], [Link]), but all were eutrophic or hypereutrophic according to IPCC classifications based on total P concentrations (IPCC, 2019). The studied wetlands were all shallow (estimated mean depth less than 5 m), where 14 out of 17 sites were smaller than 5 ha. All sites harboured hydrophytic plants characteristic of wetlands (Richardson et al., 2022), with a varying abundance of emerging aquatic vegetation such as reeds (mainly *Phragmites australis*), cattails (*Typha*), and surrounding rushes (Juncaceae) and sedges (mainly *Carex*). The shorelines along the sampled wetlands all showed characteristic wetland vegetation and waterlogged soils, further assuring their wetland characterization. By including a breadth of wetland types, we aimed to obtain a dataset covering a range of nutrient status and productivity. The sites are mainly used for wildlife hunting or grazing livestock.

### 2.1 Insect collection

To collect emerging aquatic insects, we placed two floating emergence traps (Cadmus et al., 2016) anchored approximately 50 m apart at a maximum of 2 m from the shoreline and at 0.1–2 m water depth for 72 h, which represents the emergence rate per 72 h. Trap placement was chosen to minimize the amount of floating and emergent vegetation directly beneath the trap, as vegetation may form a physical barrier hindering insect emergence. On three occasions, we lost individual traps during collection, prompting us to correct emergence rates for these sampling occasions by doubling the rate found per detected genera in the sample collected from the remaining trap at the site. Chironomids captured in the emergence traps were identified to genus level for samples from the first four sampling times, omitting the October collection as few emerging insects were captured during that period, whereas other insects were captured at very low densities in all samples and are not further discussed. In total, 43 chironomid genera were identified, with ca. 115 species present among the 5972 specimens collected. We calculated chironomid genus‐, and feeding group diversity (algae eaters, animal eaters, detritivores, omnivores and vegetation eaters) at the sample level and across all collections (total diversity) using Shannon‐Wiener diversity indexes for biodiversity‐ and community composition analyses (see divisions in Table S2). We chose functional feeding groups based on ecological relevance when considering primary production to elucidate trait‐specific effects from water temperature, nutrient (N:P) ratios and primary production due to the heterogeneous behaviours and great abundance variance among chironomid species. If the genus includes multiple feeding groups, the number of chironomids is divided equally between the feeding groups for that genus. In 10 cases where feeding group identity was unknown, the taxa were omitted from the functional community analyses.

### 2.2 Nutrient, GHG and chlorophyll‐*a* collection

On each sampling occasion, we measured water temperature and chlorophyll concentrations in situ and collected surface water and dissolved gas samples next to one of the duplicate insect collection points in each wetland. First, water samples for nutrient analysis were collected in Nalgene bottles that were kept refrigerated after collection until nutrient analysis. The analyses of total P and N were performed at the Geochemical Laboratory at the Swedish University of Agricultural Sciences, which has been SWEDAC accredited since 1992. A growing body of evidence points to the importance of nutrient stoichiometry rather than just absolute concentrations in driving ecological processes and GHG dynamics (e.g. Graeber et al., 2021), and we therefore calculated the relative stoichiometric molar N:P ratio from the measured concentrations. Second, we collected samples for analysis of dissolved GHG concentrations using the headspace method: 30 mL of water from 5 cm depth was collected into a 60‐mL syringe, and then 30 mL of ambient air one m above the wetland was also collected. The syringe was shaken vigorously for 60 s, and 15 mL of the headspace gas in the syringe were transferred to a 21‐mL glass Exetainer vial for analysis (Hope et al., 2004). CH_4_ and N_2_O in the headspace were subsequently analysed using a Clarus 500 gas chromatograph equipped with a flame ionization detector and an electron capture detector. GHG concentrations were converted from ppm to dissolved concentrations using the solubility functions from Wiesenburg and Guinasso (1979) and Weiss and Price (1980), accounting for wetland water temperature and atmospheric pressure at the time of sampling, water: air volume in the syringe, and ambient air concentration. It is important to highlight that our headspace method only gives an indication of open water GHG dynamics and does not account for plant‐mediated fluxes, which can be an important pathway for CH_4_ and N_2_O emissions (Jørgensen et al., 2011; Sebacher et al., 1985). However, our sampling approach is frequently used in studies of GHGs in small wetlands and ponds, especially when sampling a relatively large number of wetlands across regional scales (Jensen et al., 2023; Ray et al., 2023; Webb et al., 2019). Finally, we measured chlorophyll‐*a* concentrations in triplicate as a proxy for aquatic primary production using a FluoroSense™ chlorophyll probe. As in‐situ chlorophyll measurements are semi‐quantitative, where environmental conditions, organic matter, cell morphology and physiology, and light transparency may influence chlorophyll detection in algae (e.g. Kuha et al., 2020), we interpret effects on, and by, aquatic primary production with caution. In one case, the water level was too low for adequate GHG, nutrient and chlorophyll‐*a* collection, and was therefore omitted from analyses. See Table S3 for means and ranges of variables.

### 2.3 Data analysis

We modelled concentrations of chlorophyll‐*a*, CH_4_ and N_2_O, chironomid emergence rate and sample diversity using N:P ratio and water temperature as fixed factors and site as a random factor (see Table S4 for the final model setup) with lmer from the lme4 package (Bates et al., 2015). Response variables were log‐transformed to normalize residual distributions, and explanatory variables, apart from water temperature, were log‐transformed to linearize relationships. Inspection of residuals from final models confirmed that this procedure was sufficient to fulfil model assumptions. Model selection for all responses started from greatest complexity, including all fixed factors and ecologically relevant interaction terms for all models, and was reduced based on explanatory relevance and Akaike Information Criterion (AIC) scores. We modelled total chironomid emergence across all sampling using regression analysis and total diversity using Pearson's correlation against average N:P ratio, chlorophyll‐*a*, CH_4_ and N_2_O concentrations to omit seasonal variation. To examine community composition effects, we used manyglm in the mvabund‐package (Wang et al., 2022) where multivariate effects on community level are calculated by cumulative deviances from univariate responses in the separate groups. To determine effect directions on specific taxonomic groups and feeding groups, we used glmer‐models with negative binomial distributions in the lme4‐package (Bates et al., 2015). All analyses were run in R ver. 4.2.1 (R Core Team, 2022).

## 3 RESULTS

### 3.1 Relationships between nutrients, chlorophyll‐*a*, GHGs and chironomids

**FIGURE 2:**
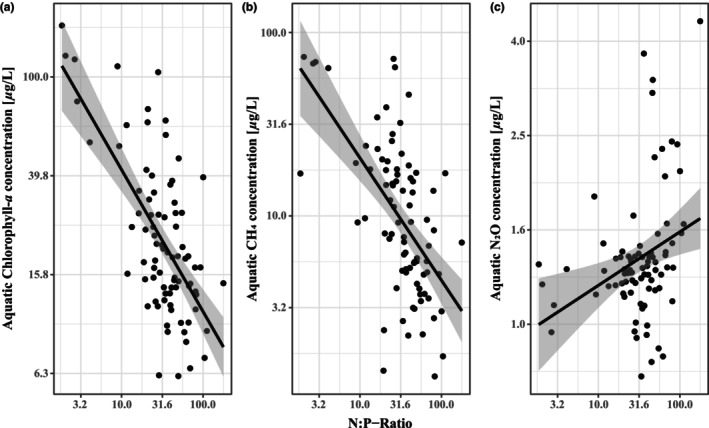
Modelled marginal response from aquatic N:P‐Ratio on (a) chlorophyll‐*a* concentration, (b) CH_4_ concentration and (c) N_2_O concentration, with corresponding linear relationship and 95% confidence intervals (CI), based on partial residuals.

**FIGURE 3:**
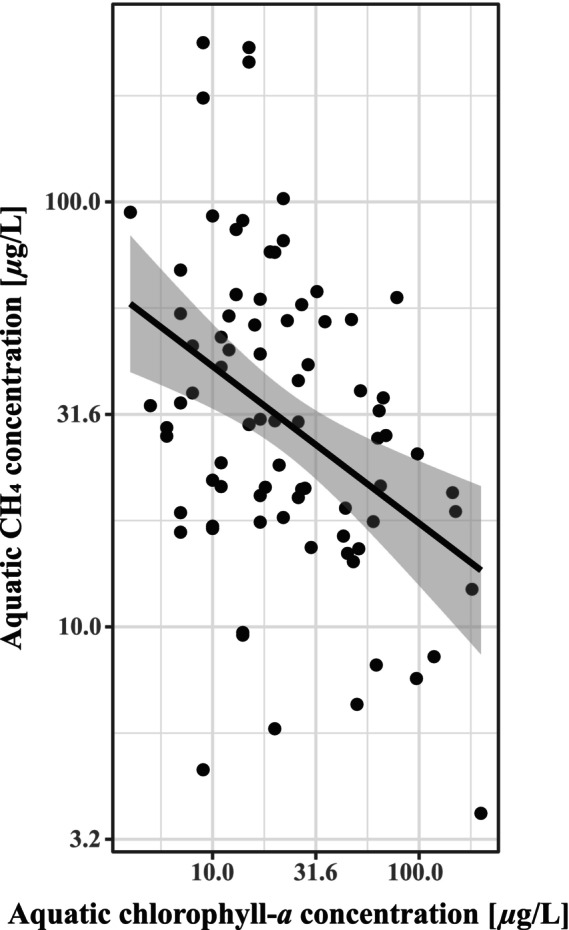
Modelled marginal response from aquatic chlorophyll‐*a* concentration on CH_4_ concentration with corresponding linear relationship and 95% CI based on partial residuals.

**FIGURE 4:**
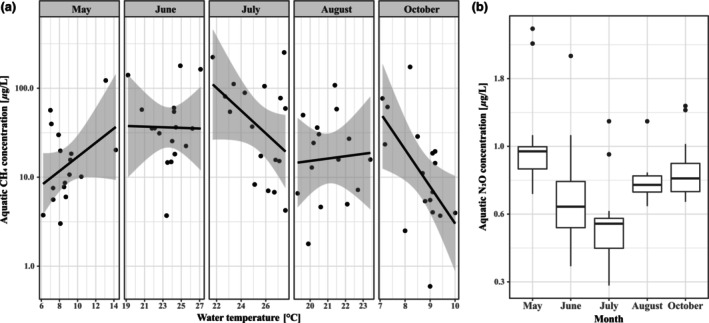
Variation in (a) CH_4_ concentration from temperature between months with linear relationship and 95% CI, and (b) N_2_O concentrations between months.

Chlorophyll‐*a* (*p* < .001, $\chi_{1}^{2}$  = 25.04) and CH_4_ (*p* < .01, $\chi_{1}^{2}$  = 10.54) concentrations decreased, whereas N_2_O concentrations increased (*p* < .01, $\chi_{1}^{2}$  = 7.86) with greater N:P ratios (Figure 2). Neither GHG was affected by N alone; however, CH_4_ increased (*p* < .05, $\chi_{1}^{2}$  = 6.22) and N_2_O decreased (*p* < .01, $\chi_{1}^{2}$  = 6.65) with greater P concentrations. CH_4_ concentrations also decreased with an increasing chlorophyll‐*a* concentration (Figure 3, *p* < .05, $\chi_{1}^{2}$  = 6.01), was higher in warmer wetlands in May and August, and lower in warmer wetlands in July and October (Figure 4a, *p* < .05, $\chi_{4}^{2}$  = 12.22). N_2_O also varied between months (*p* < .05, $\chi_{4}^{2}$  = 10.95), with greatest concentrations in May and October and lower during the summer months (Figure 4b), but was unaffected by water temperature.

**FIGURE 5:**
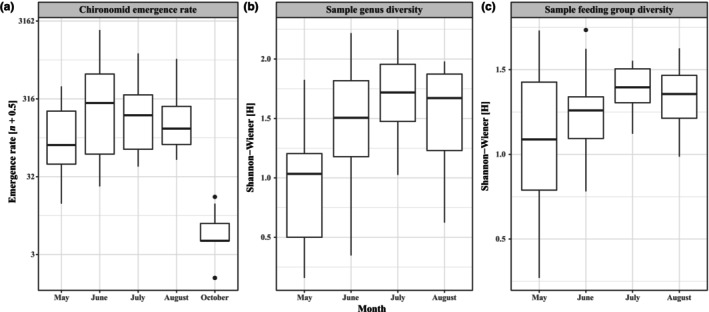
Monthly variation in (a) chironomid emergence rate, (b) sample genus diversity, and (c) sample feeding group diversity.

**FIGURE 6:**
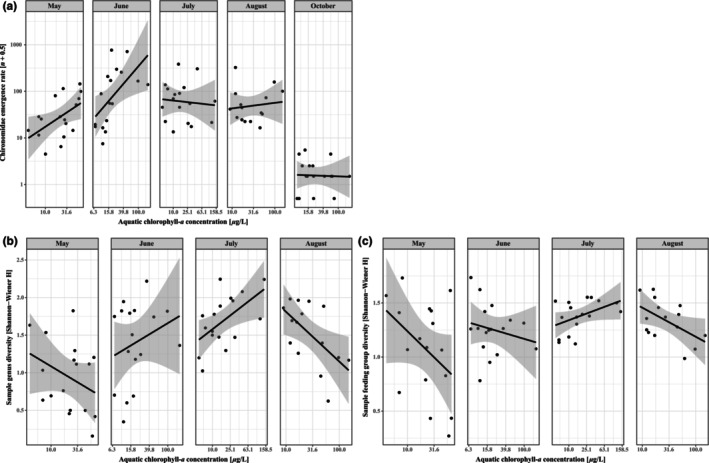
Monthly chlorophyll‐*a* effects on (a) chironomid emergence rate, (b) sample genus diversity and (c) sample feeding group diversity with corresponding linear relationship and 95% CI.

**FIGURE 7:**
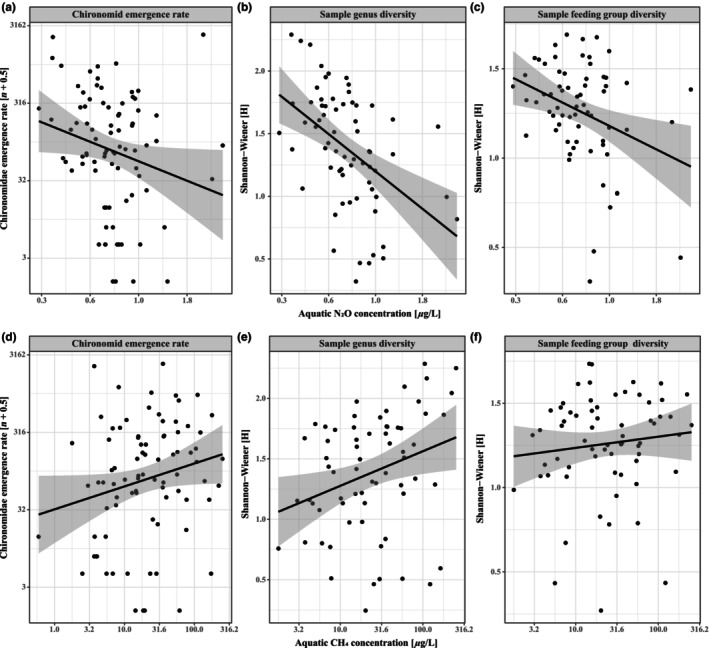
Modelled marginal response from N_2_O (a, b and c), and CH_4_ concentrations (d, e and f) on chironomid emergence rate (a and d), sample genus diversity (b and e), and sample feeding group diversity (c and f), with corresponding linear relationships and 95% CI based on partial residuals.

Chironomid emergence rates peaked during summer the months, with lower hatch rates in spring and fall (Figure 5a, *p* < .001, $\chi_{4}^{2}$  = 266.1), with a similar effect on sample genus diversity (Figure 5b, *p* < .001, $\chi_{3}^{2}$  = 35.6), and sample feeding group diversity (Figure 5c, *p* < .01, $\chi_{3}^{2}$  = 13.1). Chironomid emergence rates also responded to chlorophyll‐*a* concentrations between months (*p* < .01, $\chi_{4}^{2}$  = 18.4) where the main pattern was that wetlands with greater chlorophyll‐*a* concentrations had higher emergence rates in May and June (Figure 6a). The patterns for sample genus and feeding group diversities were more complex, with changes in different directions for different months (Figure 6, genus diversity, *p* < .01, $\chi_{3}^{2}$  = 13.5; feeding group diversity, *p* < .05, $\chi_{3}^{2}$  = 8.2). Chironomid emergence rates (*p* < .05, $\chi_{1}^{2}$  = 4.25), genus diversity (*p* < .001, $\chi_{1}^{2}$  = 14.42) and feeding group diversity (*p* < .01, $\chi_{1}^{2}$  = 7.33) correlated negatively with N_2_O concentrations (Figure 7). Genus diversity also correlated positively with CH_4_ concentration (*p* < .05, $\chi_{1}^{2}$  = 4.91); chironomid emergence rates showed a near significant positive correlation with CH_4_ concentrations (*p* = .06, $\chi_{1}^{2}$  = 3.7), whereas feeding group diversity did not vary with CH_4_ concentrations (Figure 7).

Total chironomid emergence rates per wetland, across all collections, increased with the chlorophyll‐*a* concentration (*p* < .05, *t* = 2.84, df = 15), but were unaffected by N:P ratios and uncorrelated to both GHGs. Total feeding group diversity, however, decreased with greater chlorophyll‐*a* concentrations (*p* < .05, *t* = −2.66, df = 15). Total genus diversity across seasons was also positively correlated with CH_4_ concentration (*p* < .01, *t* = 3.20, df = 15), but not with N_2_O concentrations.

### 3.2 Chironomid community composition

Water temperature (*p* < .001, Deviance = 145.9), month (*p* < .01, Dev. = 293.4), N:P ratios (*p* < .05, Dev. = 72.6) and chlorophyll‐*a* concentrations (*p* < .05, Dev. = 92.3) affected the genus composition of chironomids, but with no interactive effects. The emergence rate of *Corynoneura* (*p* < .05, Dev. = 16.6) was higher in warmer wetlands, and this was the sole univariate genus abundance response.

**FIGURE 8:**
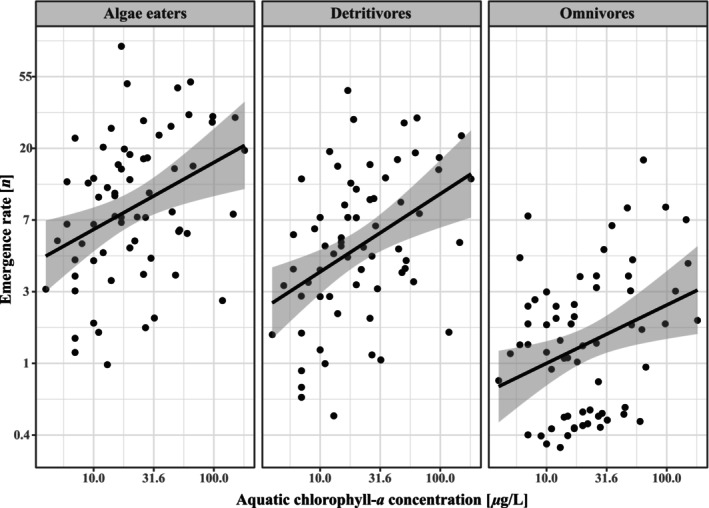
Modelled marginal response from chlorophyll‐*a* concentrations on emergence rate of algae eaters, detritivores and omnivores, with corresponding linear relationship and 95% CI based on partial residuals.

The composition of chironomid feeding group was also affected by water temperature (*p* < .01, Dev. = 37.4), month (*p* < .05, Dev. = 54.8) and chlorophyll‐*a* concentrations (*p* < .05, Dev. = 23.2), but not by N:P ratios or the interactions between the predictors. Univariate responses showed decreased abundance of algae eaters (*p* < .01, Dev. = 16.9) and detritivores (*p* < .01, Dev. = 17.2) in wetlands with higher temperatures. However, both groups also varied in abundance between months (algae eaters: *p* < .05, Dev. = 20.2; detritivores: *p* < .05, Dev. = 17.3) with the greatest emergence rates in June. Chlorophyll‐*a* concentrations also increased the abundance of both algae eaters (*p* < .05, Dev. = 7.2), detritivores (*p* < .05, Dev. = 6.8) and omnivores (*p* < .05, Dev. = 8.6) (Figure 8).

Genus composition was affected by CH_4_ concentrations (*p* < .05, Dev. = 92.9) but neither by N_2_O nor by their interaction and nor for any specific taxa. However, the interaction between CH_4_ and N_2_O affected the feeding group composition (*p* < .01, Dev. = 30.6). This interaction arose because the effect of CH_4_ affected the feeding group composition in opposite directions at low and high N_2_O concentrations. No univariate responses in any genera or feeding group were observed.

## 4 DISCUSSION

Our study is the first observational study to our knowledge to examine trade‐offs and synergies between emergence rates of chironomids and greenhouse gas production in wetland systems. Previous studies suggest the potential of such interactive effects because both rates depend on nutrient availability and primary production in wetlands (Jo et al., 2020; Malyan et al., 2022; Ramirez & Pringle, 2006). In our systems, we found that chironomid emergence rates and diversity correlated negatively to N_2_O production and (weakly) positively to CH_4_ production, suggesting that these processes are linked through some external variables. When relating GHG concentrations and chironomid variables to environmental variables, we found quite variable results between response variables but also between months. N:P ratios affected GHG concentrations but had only small effects on chironomid emergence rates or community composition. In contrast, chlorophyll‐*a* concentrations affected several measures of chironomid community composition and emergence, as well as CH_4_ but not N_2_O concentrations. The summer months also showed greater chironomid emergence rates, increased sample genus and feeding group diversity, increased abundances of certain taxa, as well as an increased production of CH_4_ and a reduced production of N_2_O. Several variables were also related to water temperature, an effect that varied between months.

The creation of wetlands favours biodiversity by increasing the production of food for aquatic groups such as fish and dragonflies (Hobson & Welch, 1995) and terrestrial predators such as spiders, birds and bats (Almenar et al., 2008; Ashley et al., 2000; Bardwell & Averill, 1997). However, biota also affects the production of greenhouse gases, particularly CH_4_. For instance, chironomids have been shown to directly utilize methane‐oxidizing bacteria (MOB) in aquatic sediments (Belle et al., 2018; Jones et al., 2008) and bioturbation caused by chironomid movements may increase oxygenation, thereby mitigating CH_4_ production (Ganglo et al., 2022, 2023). Our study is consistent with such a link between CH_4_ production and chironomid emergence rates but also suggests that the potential reduction of methane concentrations by chironomid larvae does not fully compensate for increased methane production by processes such as microbial activity. Finally, results also suggest that increased water temperatures, as would be an effect of global warming, increase chironomid productivity and diversity but may also further increase CH_4_ emissions from the wetland (e.g. Engels et al., 2020). CH_4_ concentrations likely peaked in summer because warmer temperatures enhanced methanogenesis in wetland sediments (Segers, 1998). The temperature effect on CH_4_ concentration is complex, especially in relations to methanotrophic bacterial activity (e.g. Hanson & Hanson, 1996; Lew & Glińska‐Lewczuk, 2018); however, the increased chironomid larval bioturbation in June and July may have enhanced MOB activity, thereby reducing CH_4_ production, as suggested by Ganglo et al. (2022, 2023). Lower N_2_O concentrations in the summer are at odds with some, but not all, of the constructed wetland literature and may be due to lower nitrate reduction rates in summer (Huang et al., 2013). As an approximation of how our wetlands contribute to GHG emissions, we converted GHG concentrations to fluxes using gas exchange velocities from the literature (see Peacock et al., 2021). For all wetlands, calculated CH_4_ fluxes were positive, ranging from 0.23 to 143.23 mg m^−2^ day^−1^, and thus all sites acted as sources of CH_4_. However, 7 out of 85 N_2_O measurements showed sink behaviour with negative calculated fluxes, and overall fluxes ranged from −1.85 to 35.23 μg m^−2^ h^−1^.

Dissolved CH_4_ concentrations were greatest under higher P levels, in agreement with a wide body of literature detailing enhanced methanogenesis in wetlands and waterbodies with a higher trophic state (DelSontro et al., 2018; Johansson et al., 2004). The effect of N:P ratios on CH_4_ and N_2_O concentrations acted in opposite directions. Although higher N:P has been shown to increase CH_4_ concentrations via aerobic CH_4_ production, this process mainly applies to oligotrophic systems with low P concentrations (Elser et al., 2022). In our eutrophic wetlands, a higher N:P ratio could simply result from greater levels of N, which, in inorganic forms, can have an inhibitory effect on CH_4_ production (Watson & Nedwell, 1998). The presence of nitrate would also suggest that redox conditions are not reduced enough for optimal CH_4_ production (Jugsujinda et al., 1995) and, furthermore, nitrate can be used for anaerobic CH_4_ oxidation (Raghoebarsing et al., 2006). Similarly, increasing total N may result in greater N_2_O concentrations due to the greater availability of substrates for nitrification and denitrification (Audet et al., 2020). A lower N:P could arise due to P being more plentiful, which would lead to N depletion, N limitation, and then N_2_O consumption, whilst the more reduced conditions would favour CH_4_ production. Chlorophyll‐*a* concentrations decreased with greater N:P ratios, suggesting that the phytoplankton benefitted from elevated P concentrations relative to N, in agreement with previous findings for eutrophic waterbodies (Liang et al., 2020). Chlorophyll‐*a* concentrations, in turn, were surprisingly negatively related to CH_4_ concentrations. The general assumption in wetlands, ponds and lakes is that chlorophyll and CH_4_ are positively correlated (Badiou et al., 2019; DelSontro et al., 2018), although sometimes no relationship is found between the two (Bastviken et al., 2004). An explanation for our negative relationship is that it is not causal and instead arises coincidentally because N:P significantly drives both CH_4_ and chlorophyll via the aforementioned mechanisms. As such, chlorophyll‐*a* is likely not a good measure of wetland productivity in our systems, and instead, sediment organic carbon concentrations will drive substrate availability for methanogenesis. In these systems, plant‐mediated transport (which we did not measure) will also play an important role in net wetland CH_4_ emissions (Jørgensen et al., 2011; Sebacher et al., 1985), and future studies should incorporate measurements from open water and vegetated areas in order to quantify the net wetland emission.

The effects of chlorophyll concentrations by month on chironomid emergence and diversity corroborate previous findings where food availability and quality are essential to chironomid productivity (e.g. De Haas et al., 2006; Signa et al., 2015). Chlorophyll concentrations seem to be a driving factor on emergence rates mainly in May, June and August, whereas taxonomic and functional diversity increased with greater chlorophyll concentrations in June (taxonomic) and July (both diversities) and decreased with greater concentrations in May and August, where total chironomid emergence also increased with greater chlorophyll concentrations. As chlorophyll concentrations did not vary significantly between months, emerging chironomids may be driven by food availability in spring, with the same response in those emerging in later summer. Taxonomic diversity increased considerably between May and June (see Figure 5), suggesting that specialist genera emerging in spring require greater autochthonous food concentrations for development (e.g. Berg & Hellenthal, 1992) that may be supplied by littoral phytoplankton, thereby dominating the spring community abundance. Genera emerging later alleviates this dominance as more niches open and phenology progresses, allowing taxonomic diversity to increase during the summer months. The abundance of algae feeding chironomids, along with detritivores and omnivores, was directly related to higher chlorophyll concentrations, which further corroborates the importance of ample food supplies for chironomid emergence since these groups compose ~80% of sampled chironomids. As algae eaters utilize phytoplankton as their main food source, increasing chlorophyll‐*a* concentrations from increased P suggests a bottom‐up cascading effect. As increased phytoplankton production simultaneously would increase detritus, it makes sense that detritivores are also positively related to increased chlorophyll‐*a* levels, whilst omnivores utilize a wide range of foods and thus benefit from phytoplankton production directly or indirectly by elevating other food sources that omnivores then consume. Total feeding group diversity also decreased with greater chlorophyll concentrations, suggesting that specific feeding groups relate to chlorophyll concentrations, thereby limiting diversity between them. As such, elevated aquatic primary production may be driving wetland biodiversity support for terrestrial predators feeding on chironomids.

The other main effect on the chironomid community was water temperature. The emergence of algae eaters and detritivores both decreased with increasing temperatures when controlling for season, whereas higher temperatures increased genus diversity. These findings suggest that water temperature is a main driving factor in the functionality of the chironomid community (see also Eggermont & Heiri, 2012; Marziali & Rossaro, 2013). A similar conclusion was made by Engels et al. (2020), who showed that increased temperatures in temperate regions during climate change events drove chironomid diversity. However, seasonality is perhaps even more important, directly or indirectly affected by temperature, where a higher diversity and community complexity are seen during the summer months. Phenology is important to all insects, including chironomids (Doria et al., 2022). Still, factors such as, for example oxygen levels (e.g. Villamarin et al., 2021) and abundance of fish (e.g. Nieoczym et al., 2020) have also been shown to affect diversity and abundance in chironomids. As such, water temperature is likely a significant factor in governing chironomid communities, however, there are several other factors also regulating chironomid emergence which were not measured in the present study, including water depth and sediment conditions (e.g. Chen et al., 2014; Duan et al., 2009), and purpose of creation.

To conclude, our study corroborates previously suggested trade‐offs between chironomid production and diversity to CH_4_ production and shows that their production is linked by the same mechanisms in these habitats. However, the same trade‐off was not observed for N_2_O. As wetland biodiversity support is generally considered to rely on the elevated productivity rate, as also indicated in this study, it is clear that such aquatic‐terrestrial energy fluxes also come with the drawback of producing CH_4_. Wetlands are heralded as natural solutions to problems caused by expanding human enterprises, where increasing biodiversity can be seen as a mitigating factor for adverse effects. However, it is evident that the benefit of one aspect may come with the detriment of another.

## AUTHOR CONTRIBUTIONS

**David Åhlén:** Conceptualization (lead); data curation (lead); formal analysis (equal); funding acquisition (equal); investigation (equal); methodology (equal); project administration (equal); visualization (equal); writing – original draft (lead); writing – review and editing (equal). **Mike Peacock:** Conceptualization (equal); data curation (equal); funding acquisition (equal); investigation (equal); methodology (equal); resources (equal); validation (equal); writing – original draft (equal); writing – review and editing (equal). **Yngve Brodin:** Data curation (equal); investigation (equal); methodology (equal); validation (equal); writing – original draft (supporting); writing – review and editing (equal). **Peter A. Hambäck:** Conceptualization (equal); data curation (equal); formal analysis (equal); funding acquisition (equal); investigation (equal); methodology (equal); project administration (equal); supervision (equal); validation (equal); writing – original draft (equal); writing – review and editing (equal).

## CONFLICT OF INTEREST STATEMENT

The authors declare no conflicts of interest. Any opinion, conclusion and recommendation in the article are those of the authors.

## Supporting information


Figure S1

Figure S2

Figure S3

Figure S4

Figure S5

Figure S6

Figure S7

Figure S8

Figure S9

Figure S10

Figure S11

Figure S12

Figure S13

Figure S14

Figure S15

Figure S16

Figure S17

Table S1

Table S2

Table S3

Table S4

Data S1

## Data Availability

The data that support the findings of this study are openly available in Dryad at https://doi.org/10.5061/dryad.qrfj6q5mb (Åhlén et al., 2023).
