# Supplementary figures and images for: Wetland productivity determines trade‐off between biodiversity support and greenhouse gas production
Source: Ecol Evol. 2023 Oct 20;13(10):e10619. doi: 10.1002/ece3.10619 (PMC10587742; doi:10.1002/ece3.10619)

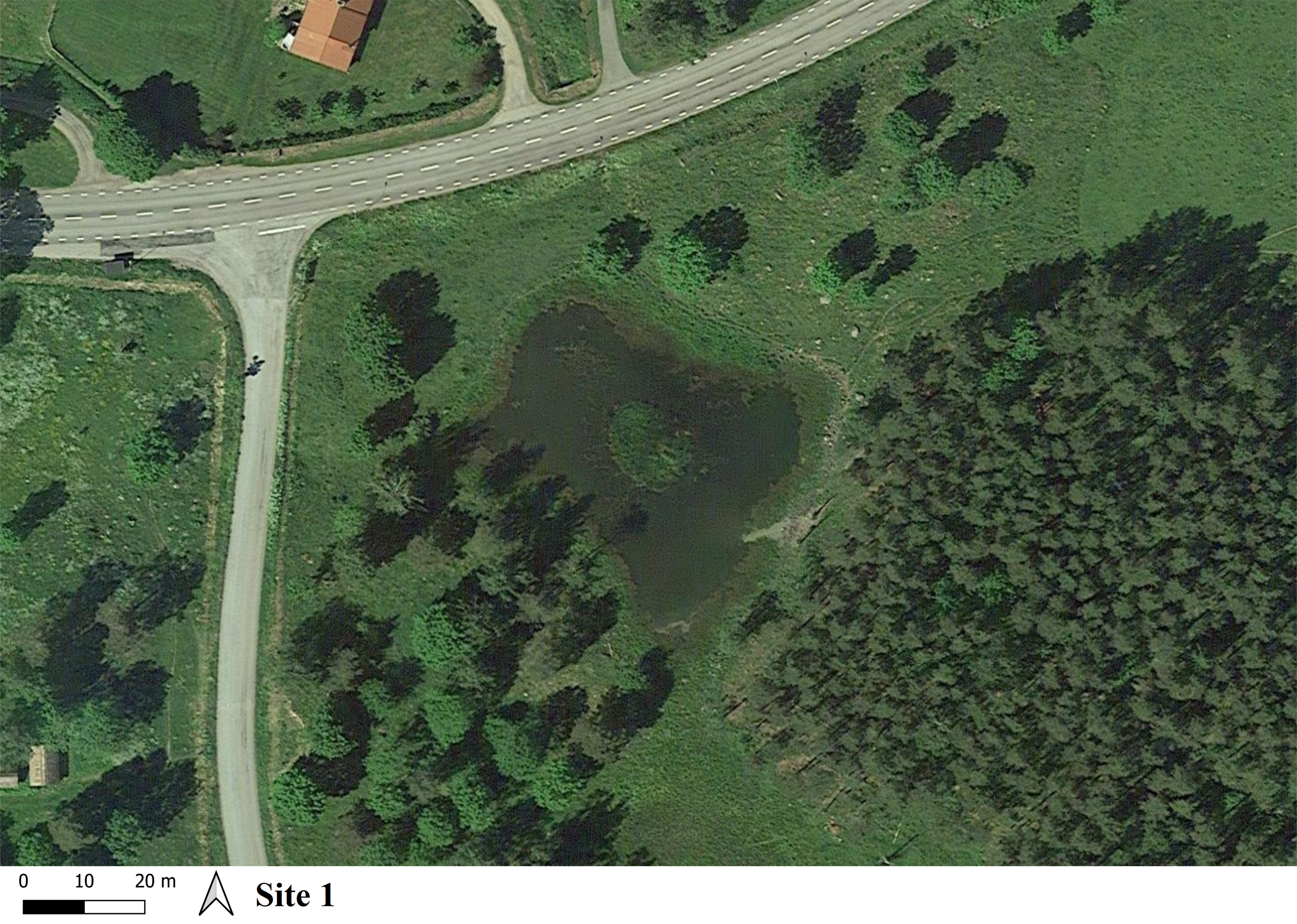

Supplement: Supplementary file 1 — Figure S1 [file ECE3-13-e10619-s004.png]

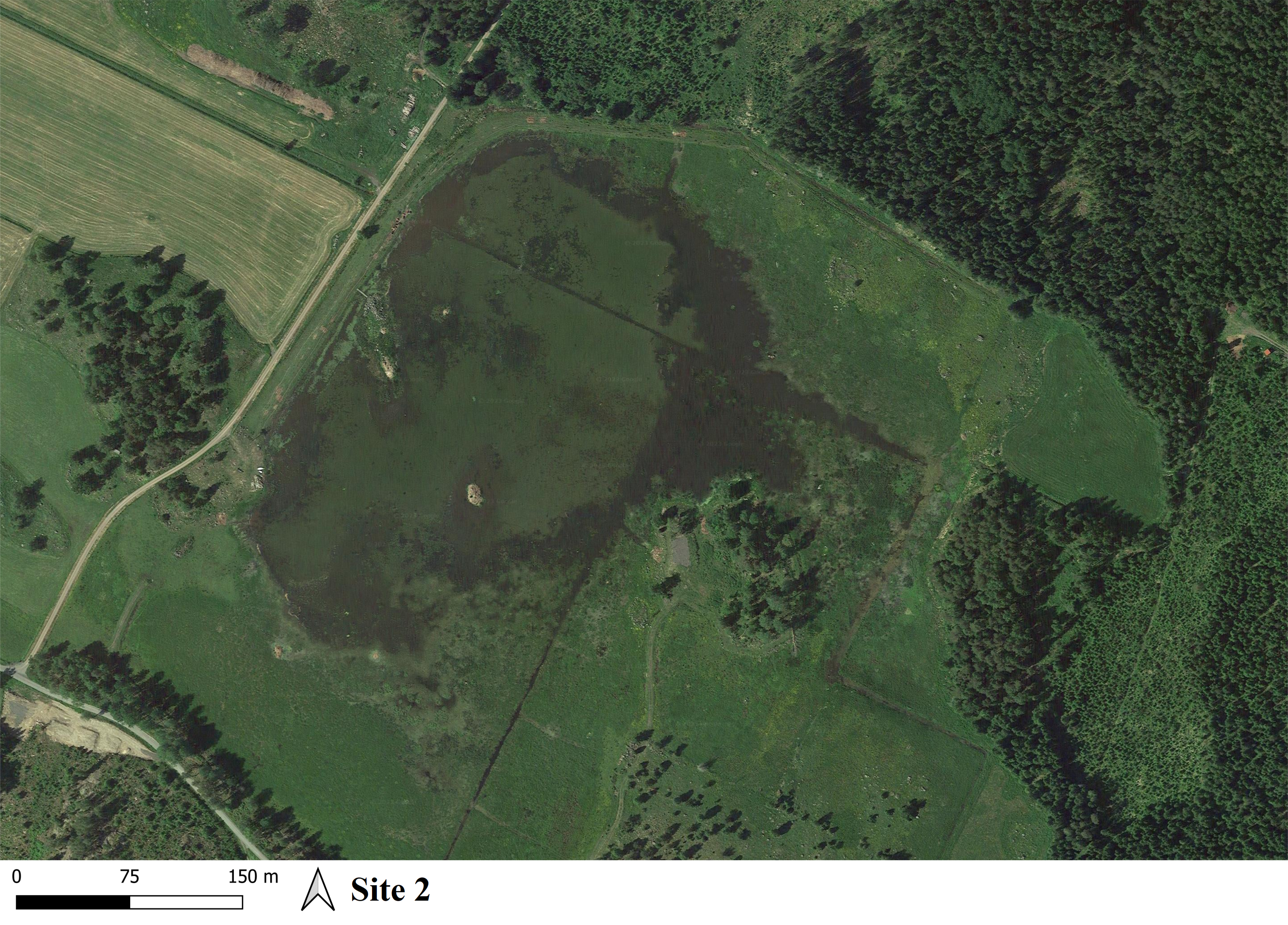

Supplement: Supplementary file 2 — Figure S2 [file ECE3-13-e10619-s002.png]

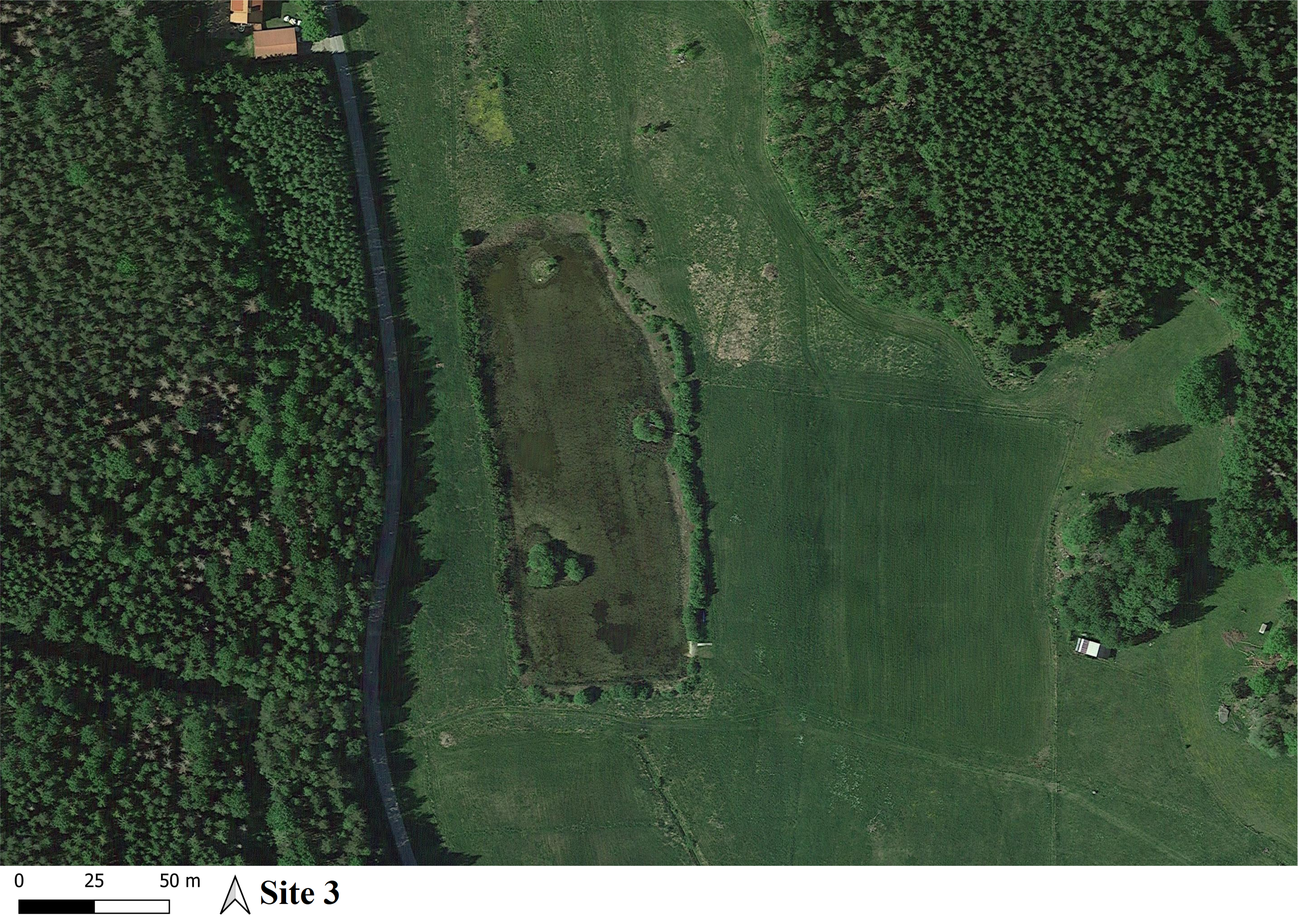

Supplement: Supplementary file 3 — Figure S3 [file ECE3-13-e10619-s014.png]

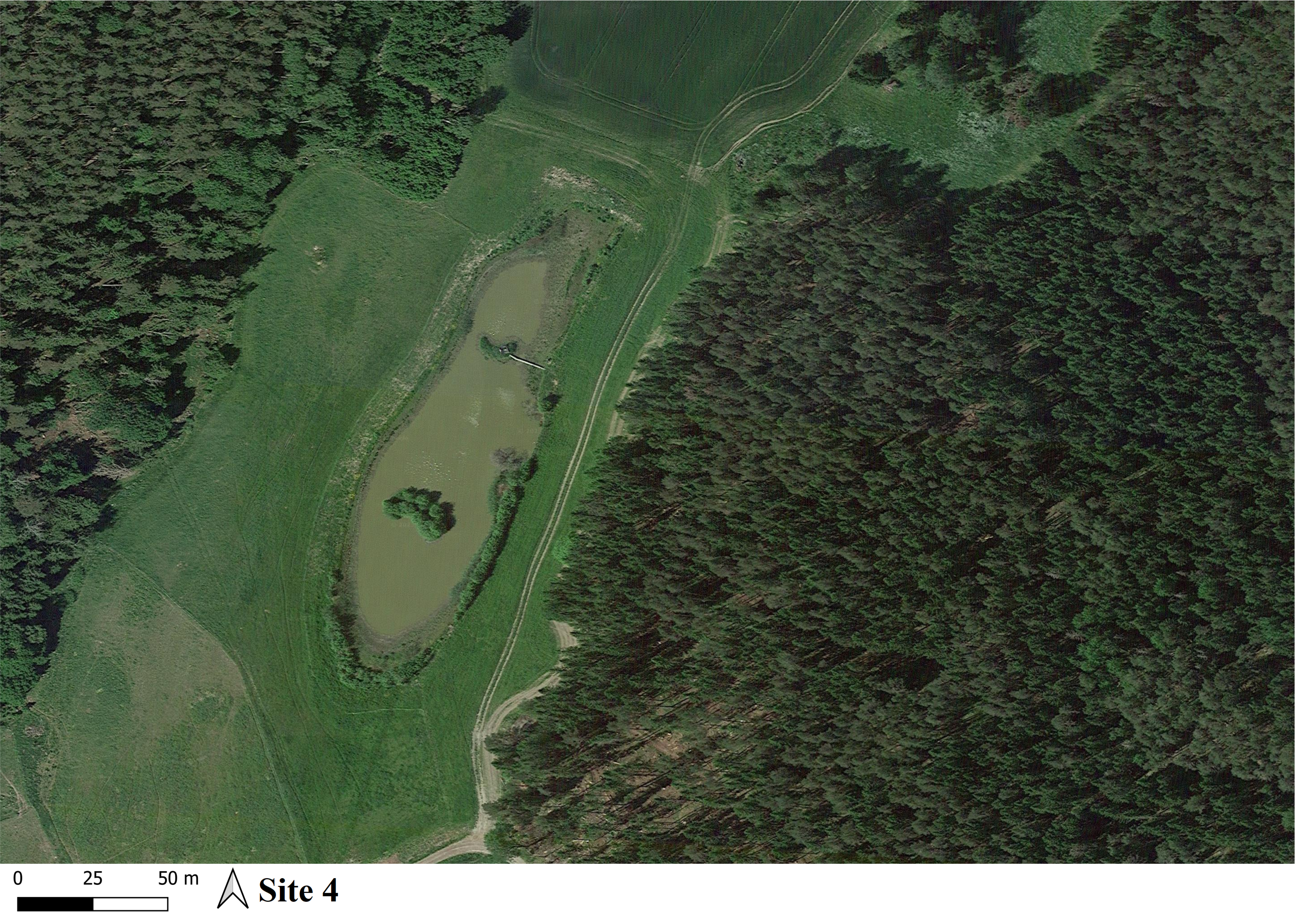

Supplement: Supplementary file 4 — Figure S4 [file ECE3-13-e10619-s011.png]

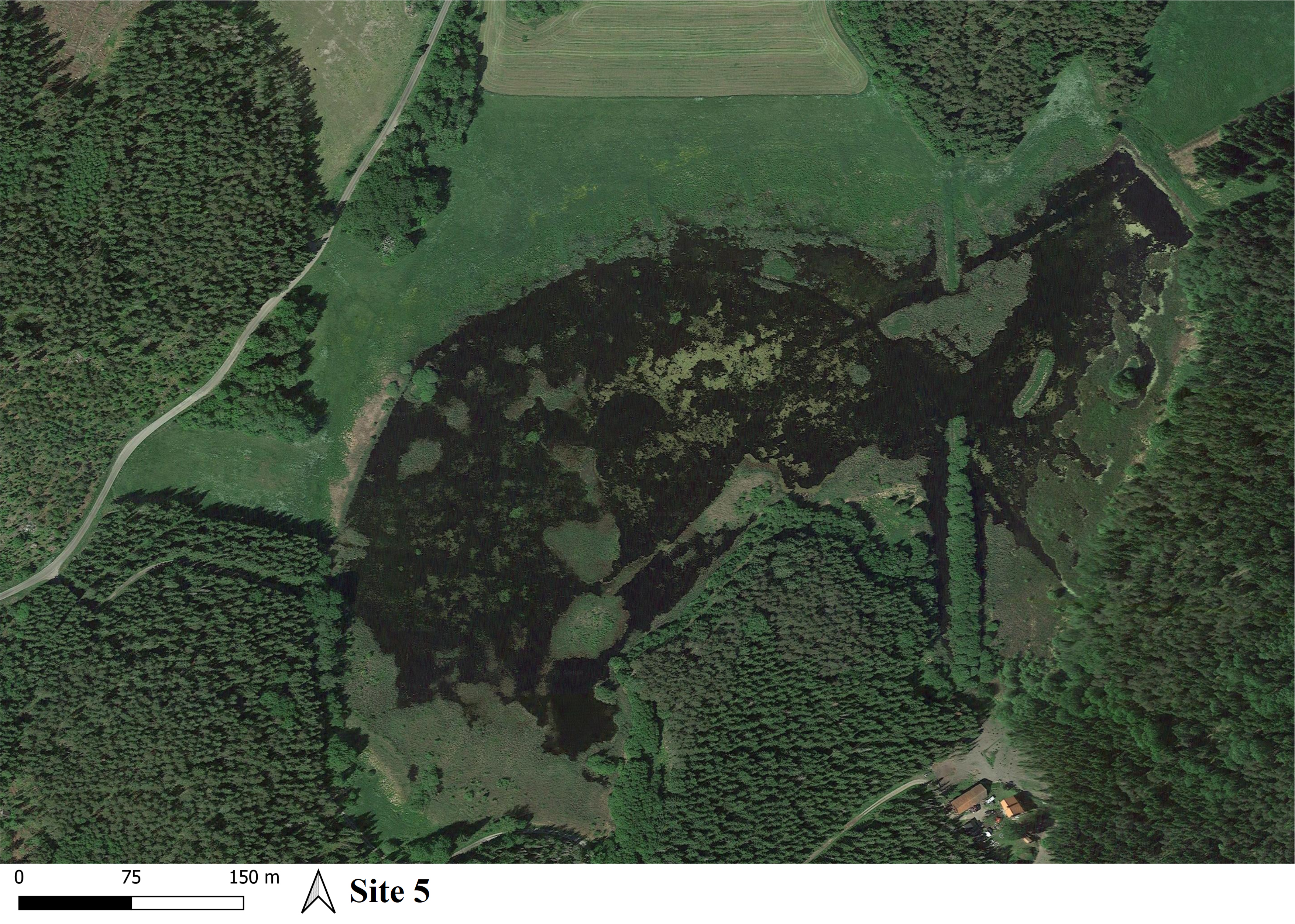

Supplement: Supplementary file 5 — Figure S5 [file ECE3-13-e10619-s017.png]

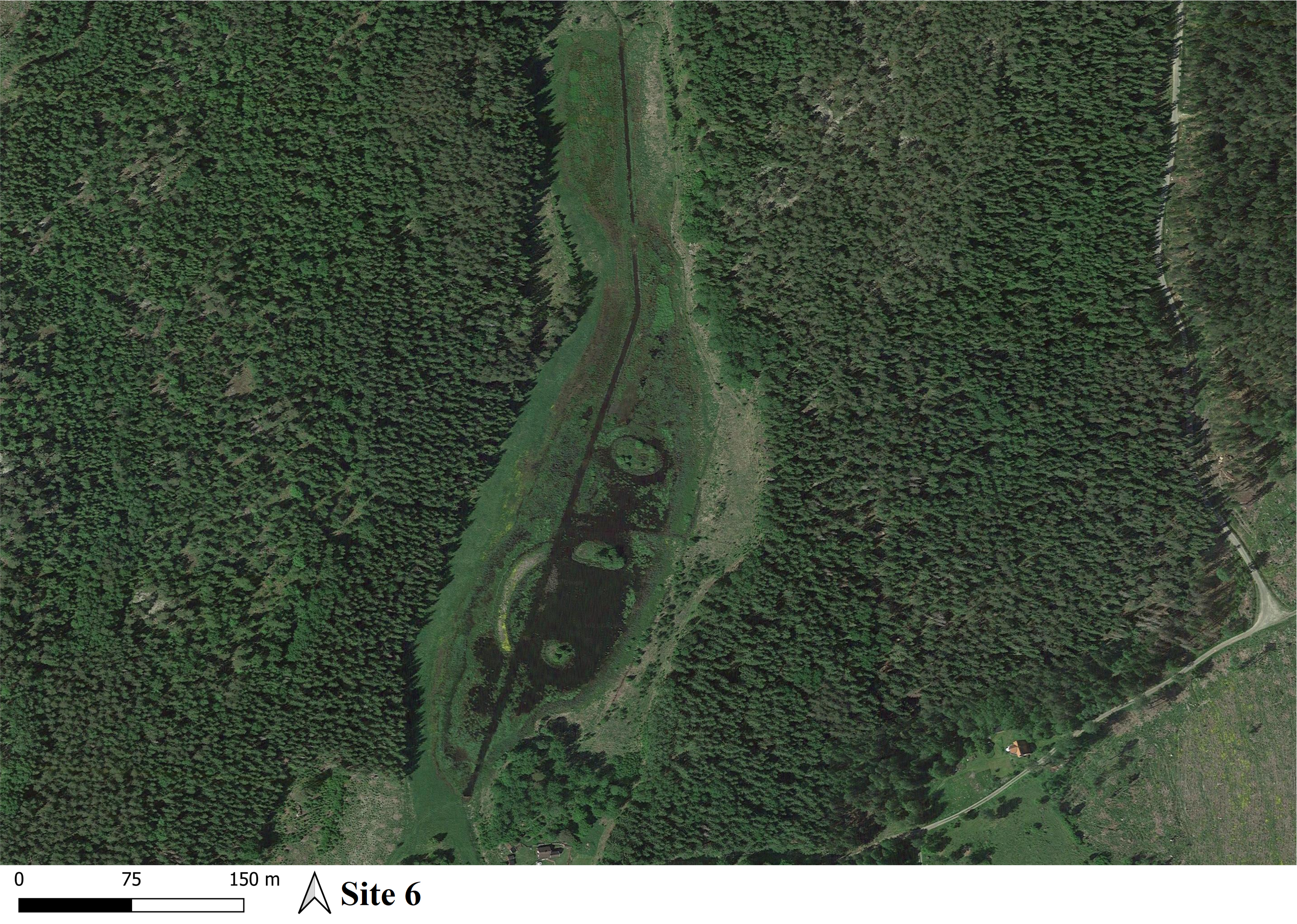

Supplement: Supplementary file 6 — Figure S6 [file ECE3-13-e10619-s022.png]

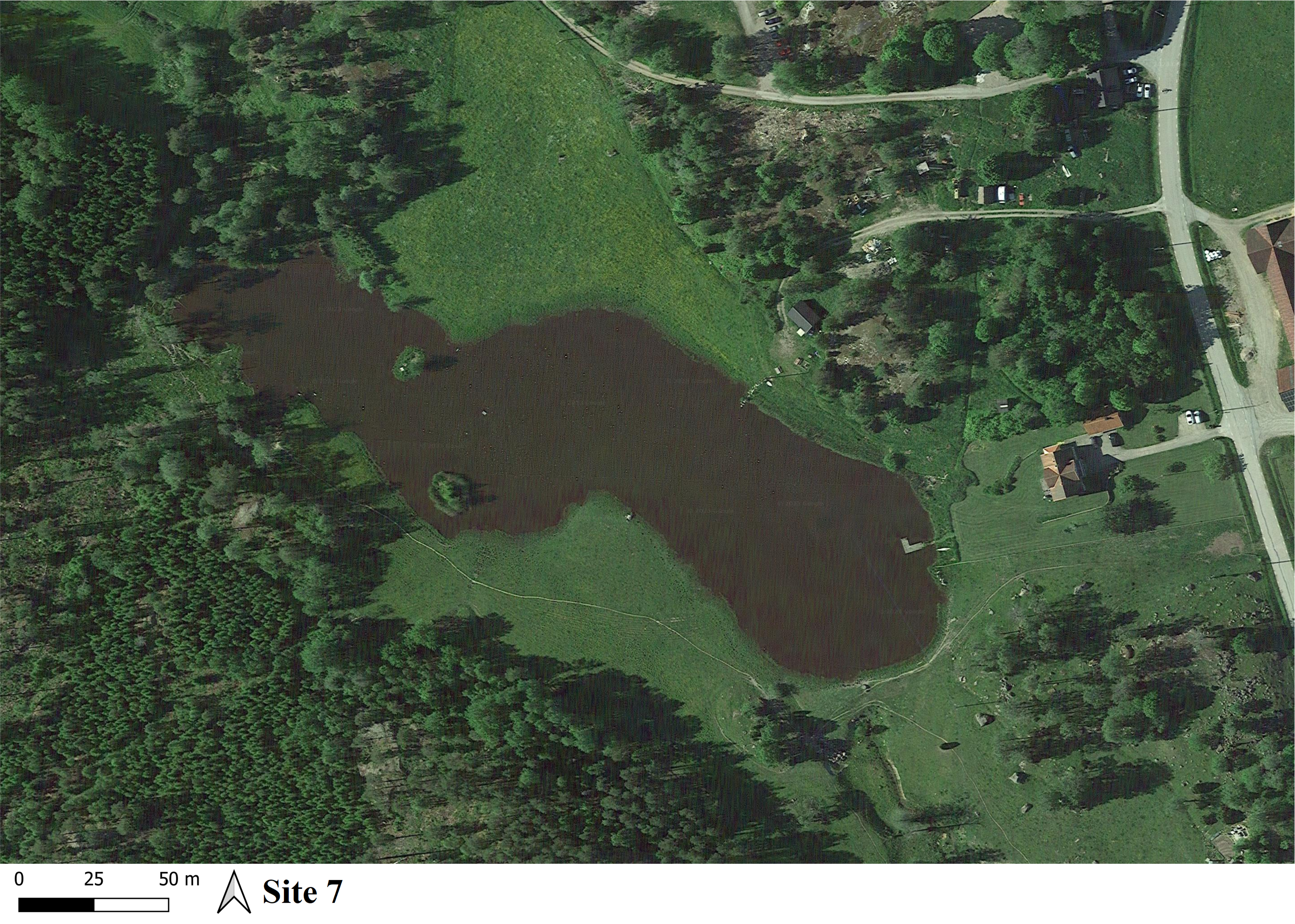

Supplement: Supplementary file 7 — Figure S7 [file ECE3-13-e10619-s007.png]

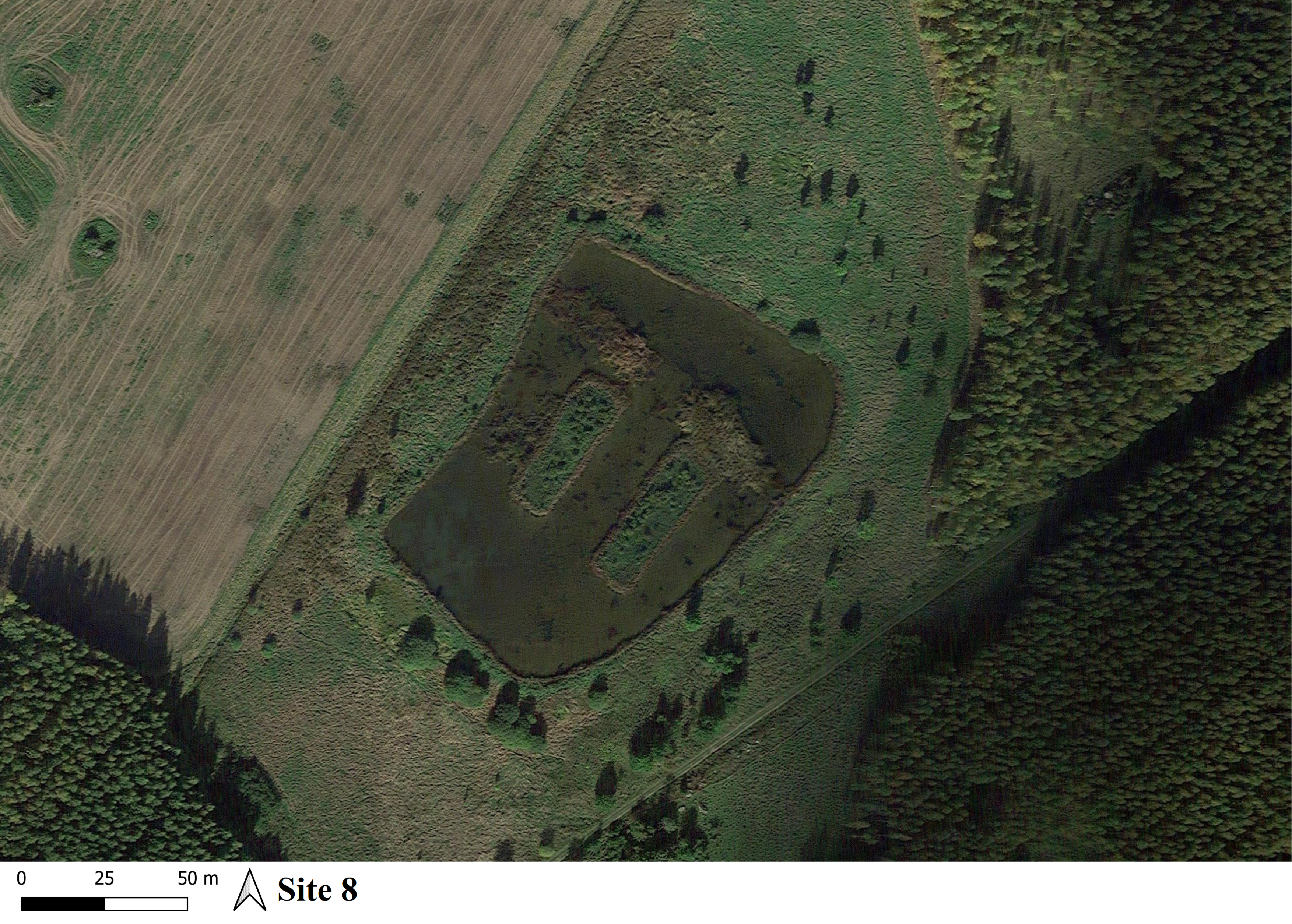

Supplement: Supplementary file 8 — Figure S8 [file ECE3-13-e10619-s012.png]

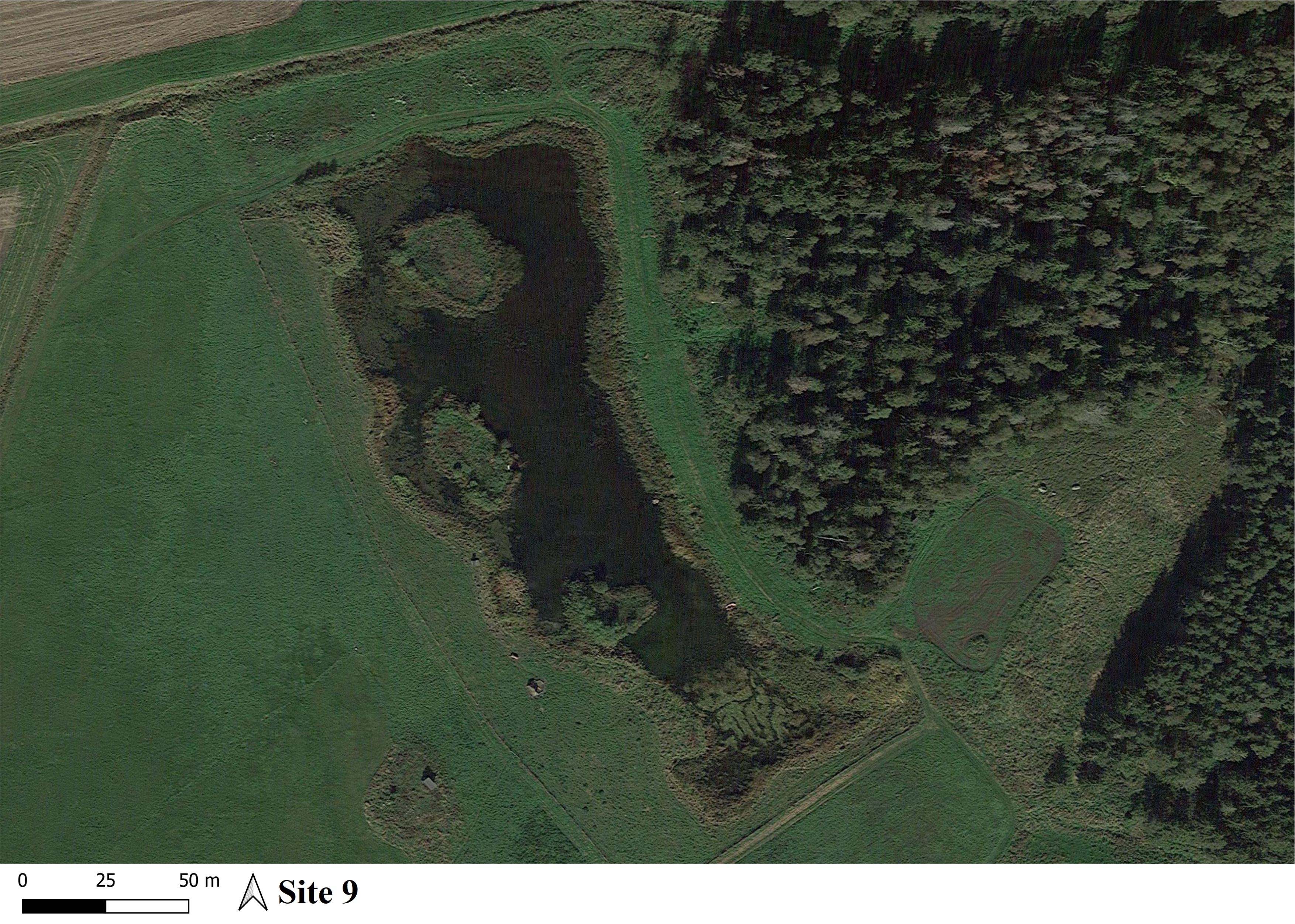

Supplement: Supplementary file 9 — Figure S9 [file ECE3-13-e10619-s003.png]

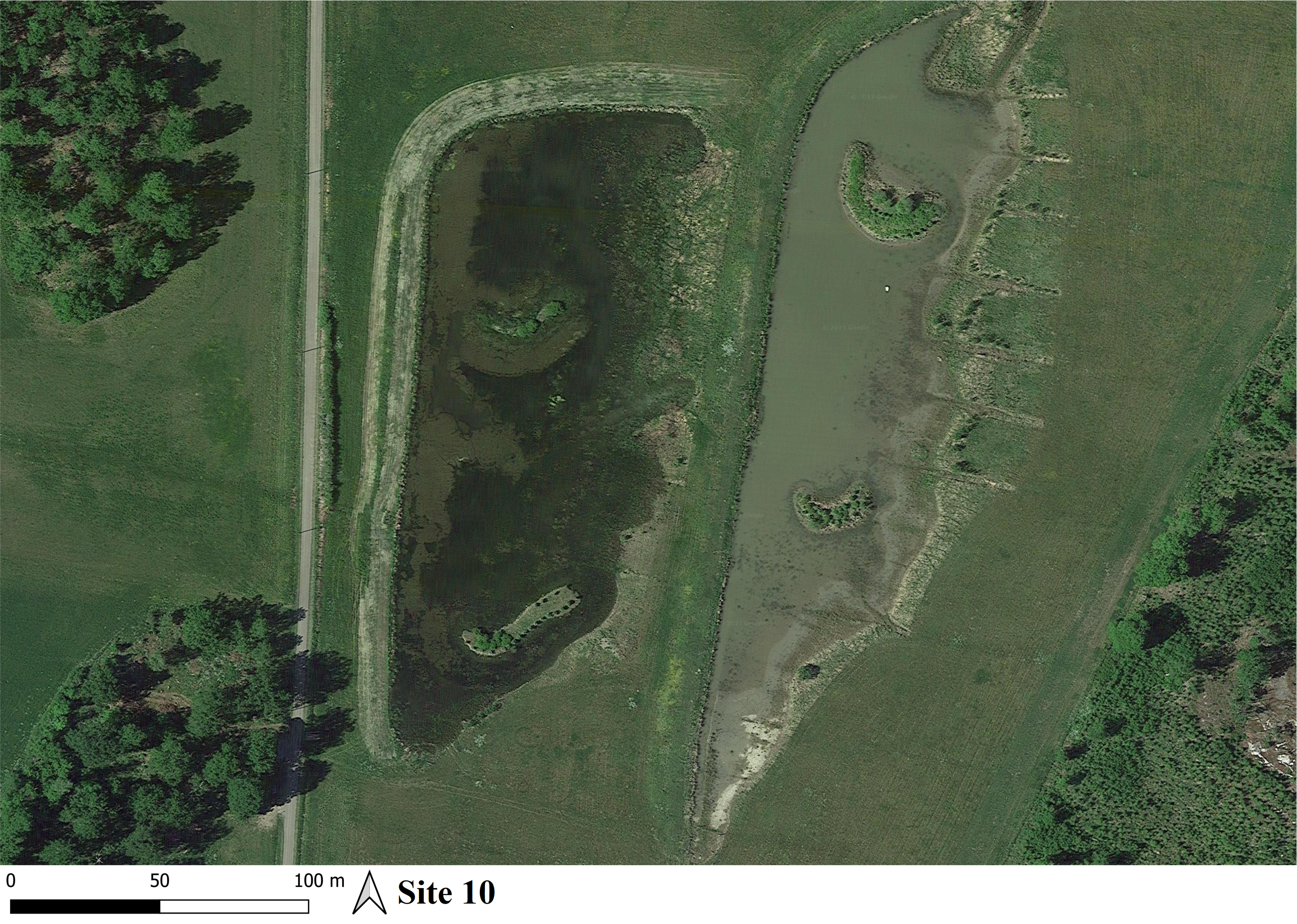

Supplement: Supplementary file 10 — Figure S10 [file ECE3-13-e10619-s010.png]

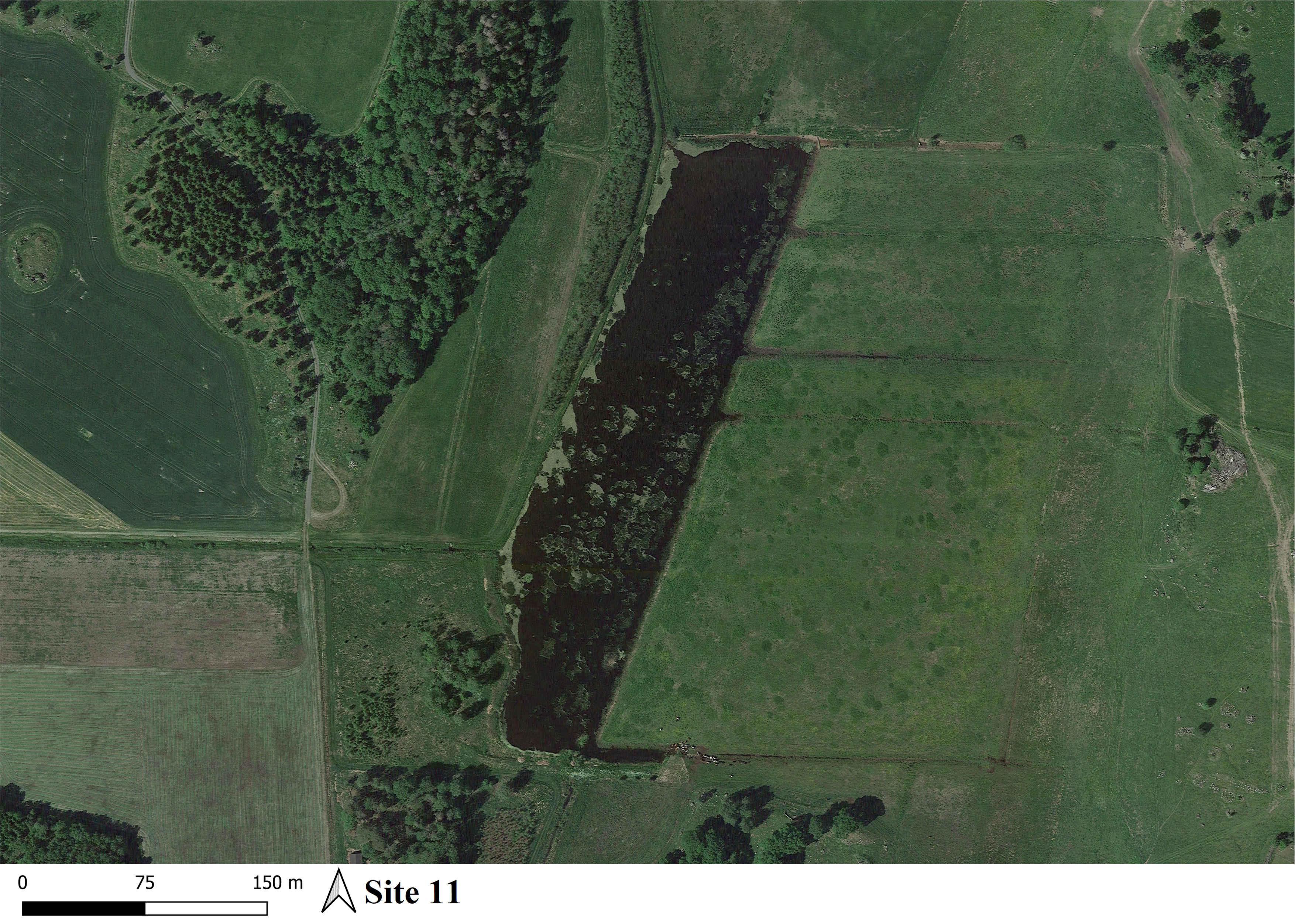

Supplement: Supplementary file 11 — Figure S11 [file ECE3-13-e10619-s019.png]

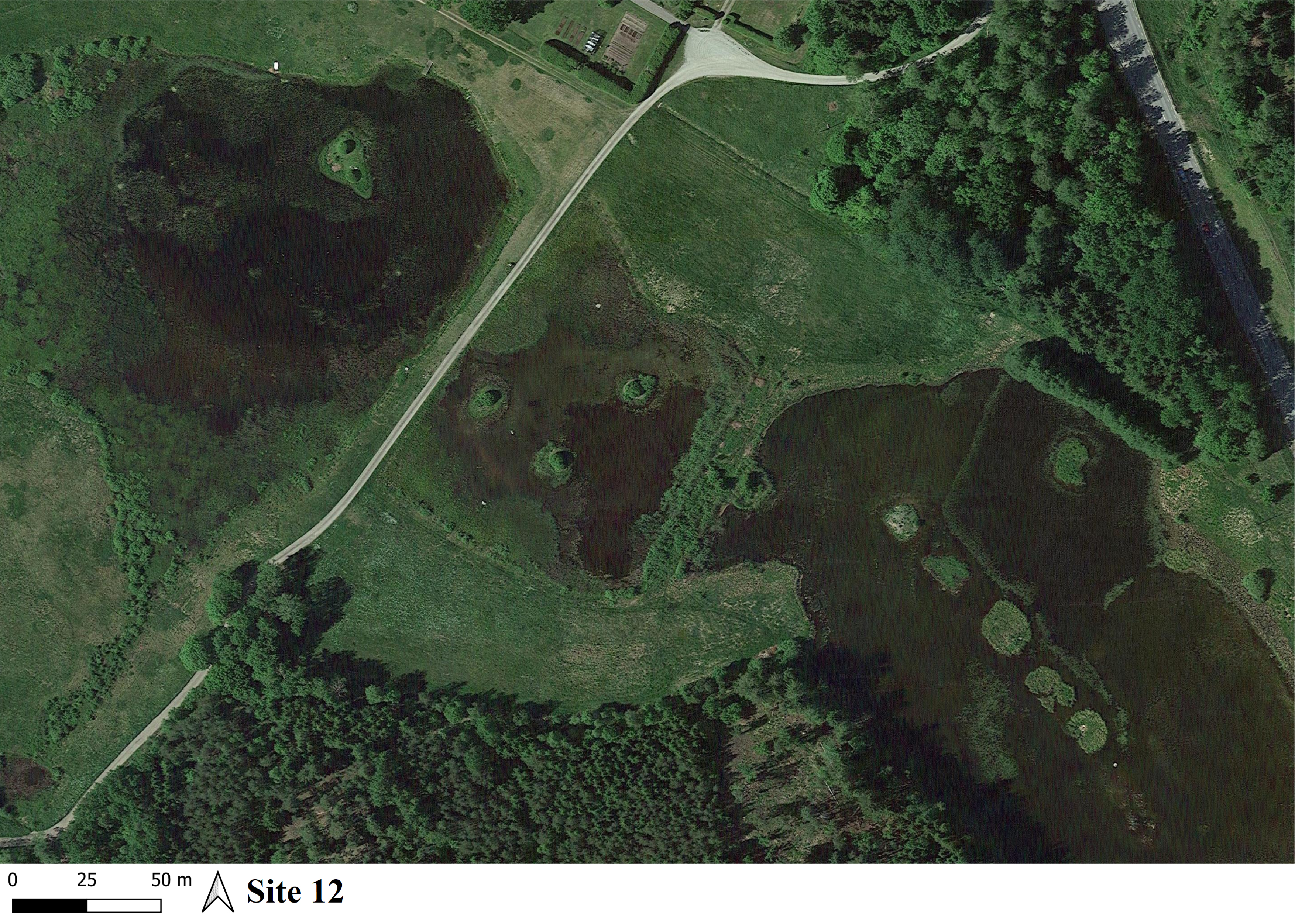

Supplement: Supplementary file 12 — Figure S12 [file ECE3-13-e10619-s015.png]

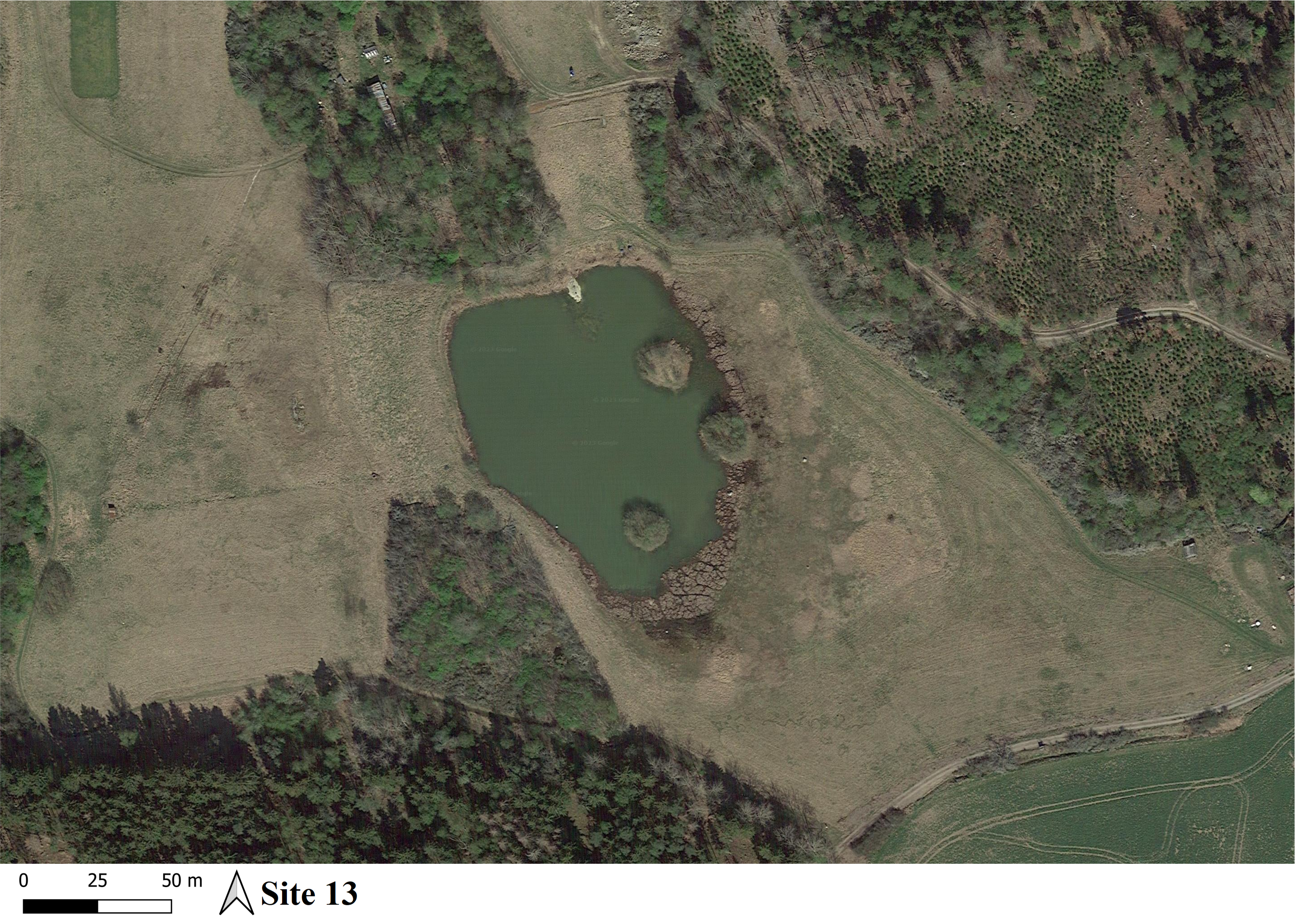

Supplement: Supplementary file 13 — Figure S13 [file ECE3-13-e10619-s005.png]

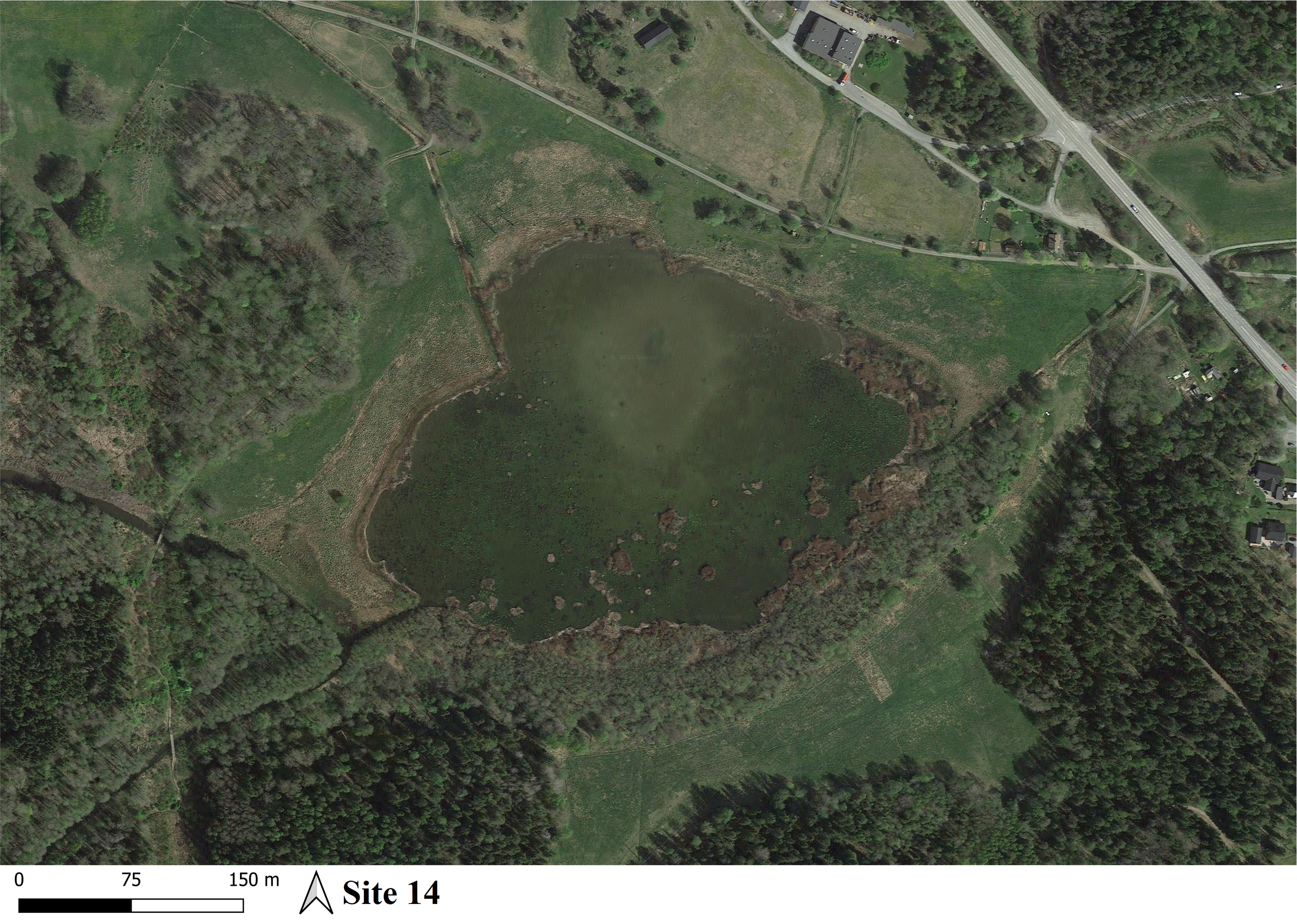

Supplement: Supplementary file 14 — Figure S14 [file ECE3-13-e10619-s001.png]

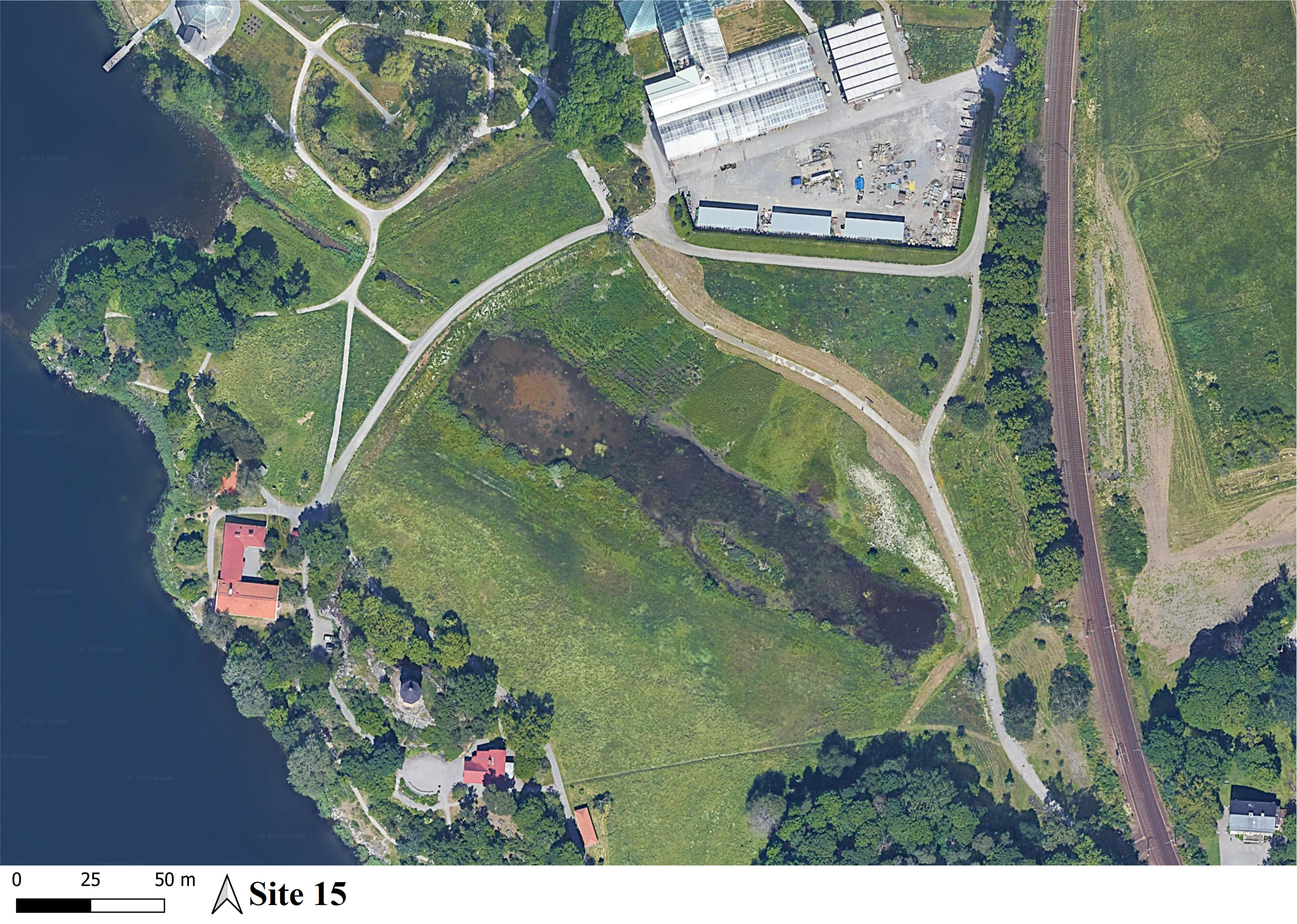

Supplement: Supplementary file 15 — Figure S15 [file ECE3-13-e10619-s013.png]

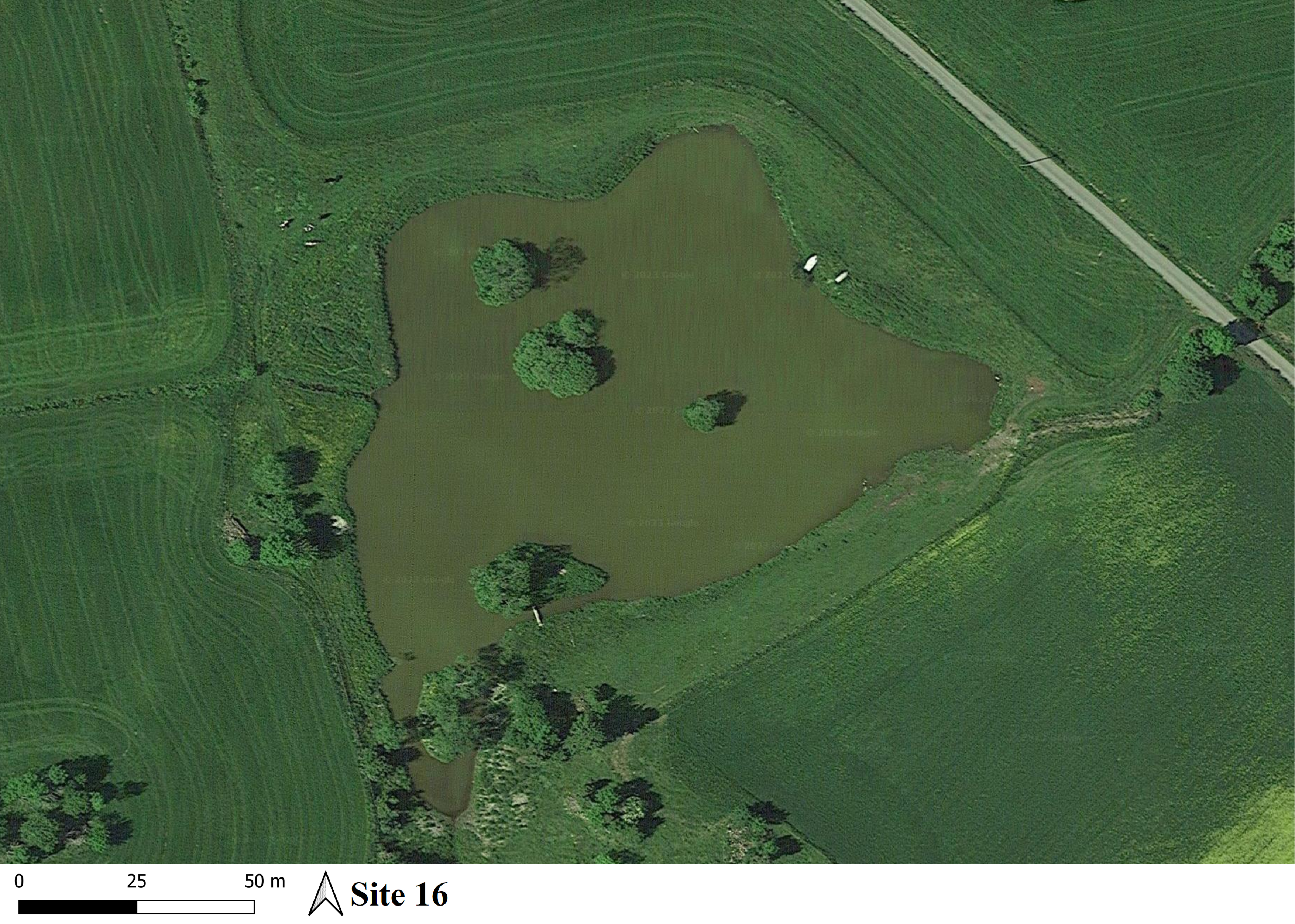

Supplement: Supplementary file 16 — Figure S16 [file ECE3-13-e10619-s016.png]

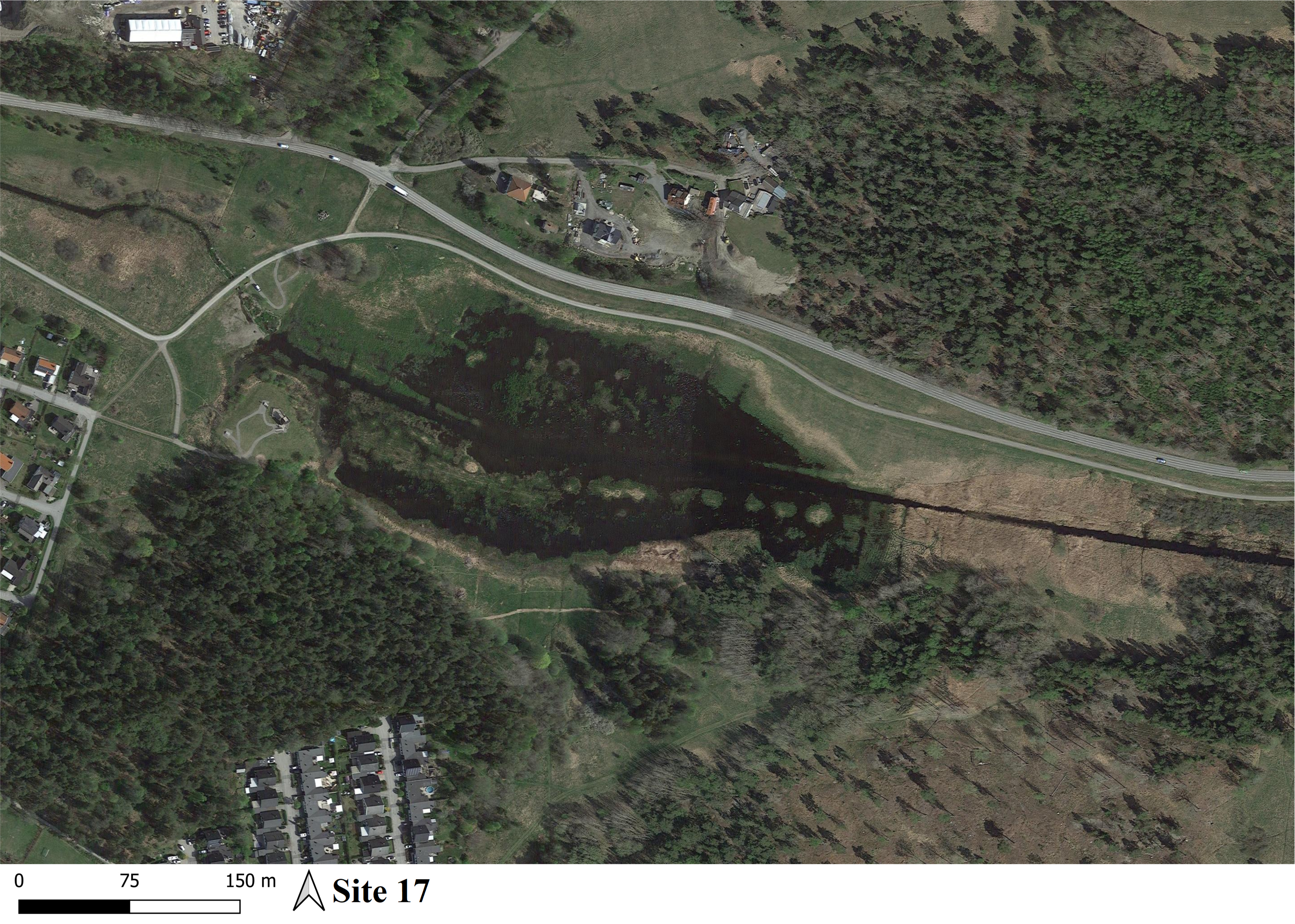

Supplement: Supplementary file 17 — Figure S17 [file ECE3-13-e10619-s006.png]
