# Supplementary material for: Wetland productivity determines trade‐off between biodiversity support and greenhouse gas production
Source: Ecol Evol. 2023 Oct 20;13(10):e10619. doi: 10.1002/ece3.10619 (PMC10587742; doi:10.1002/ece3.10619)
Supplement: Supplementary file 19 — Table S2 [file ECE3-13-e10619-s018.zip › Citations.docx]

Appendix: Sampled location information, linear mixed-effects model setups and abundance and division of chironomid genera into feeding groups

Here, we present the general site characteristics (Table S1) and aerial photographs of all sites (Figures S1–S17). We also present how abundances per genera were divided into feeding groups (Table S2) and the references used for the division, together with means and ranges of variables used for linear mixed-effects model analyses (Table S3), and their final model formats (Table S4).

Citations in appendix

1. Thienemann A. 1954: Chironomus, Leben, Verbreitung und wirtschaftliche Bedeutung der Chironomiden. - Die Binnengewässer 20: 1–834.

2. Walshe B.M. 1951a: The feeding habits of certain chironomid larvae (subfamily Tendipedinae). - The Proceedings of the Royal Entomological Society of London 121: 63–79.

3. Armitage P.D. 1968: Some notes on the food of the chironomid larvae of a shallow woodland lake in South Finland. – Annales Zoologici Fennici 5: 6–13.

4. Armitage P.D. 1974: Some aspects of the ecology of the Tanypodinae and other less common species of Chironomidae in Lake Kuusijärvi, South Finland. – Entomologisk Tidskrift 95: 13–17.

5. Zavrel J. 1928: Die Jugendstadien der Tribus Corynoneurariae. – Archiv für Hydrobiogie 19: 651–665.

6. Lellak J. 1970: Key to the determination of chironomid larvae of the family Chironomidae.

7. Armitage P.D. 1970: The Tanytarsini (Diptera, Chironomidae) of a shallow woodland lake in South Finland, with special reference to the effects of winters conditions. – Annales Zoologici Fennici 7: 313–322.

8. Cranston P.S. 1982: A key to the larvae of the British Orthocladiinae (Chironomidae). – Freshwater Biological Association Scientific Publication 45: 1–152.

9. Lenz F. Neue Beobachtungen zur Biologie der Jugendstadien der Tendipedinengattung Parachironomus Lenz. - Zool. Anz. 147: 95–111.

10. Fjellheim A. & Raddum G.G. 1988: Birch leaf processing macroinvertebrates in an acidified lake subjected to liming. - Hydrobiologia 157: 89–94.

11. Ward A.F. & Williams D.D. 1986: Longitudinal zonation and food of larval chironomids (Insecta: Diptera) along the course of a river in temperata Canada. - Holarctic Ecology 9: 48–57.

12. Barthelmes D. 1966: Zur Verbreitung und Bedeutung räuberischer Dipterenlarven im Karpfenteichen. - Verhandlungen des Internationalen Vereinigung für Limnologie 16: 1377–1382.

13. Olafsson J.S. 1992: A comparative study on mouthpart morphology of certain larvae of Chironomini (Diptera: Chironomidae) with reference to the larval feeding habits. - J Zool. London. 228: 183–204.

14. Moller-Pillot H.K.M. 1984: De larven der Nederlandse Chironomidae (Diptera). Inleiding, Tanypodinae & Chironomini. - Nederlandse Faunistische Mededelingen 1A: 1–278.

15. Belyavskaya L.I. 1956: On the feeding of Anatopynia varia F larvae [in Russian]. - Trudy Sarat. Otdel. Vniorch 4: 192–197.

16. Roback S.S. 1969: Notes on the food of Tanypodinae larvae. -Ent. News 80: 13–18.

17. Konstantinov A.S. 1961: Feeding in some predatory chironomid larvae [in Russian]. - Vop. Ichthiologii 20: 570–582.

18. Fittkau E.J. & Roback S.S. 1983: The larvae of Tanypodinae (Diptera: Chironomidae) of the Holarctic region - Keys and diagnosis. In: Chironomidae of the Holarctic region - Keys and diagnosis. Part 1. Larvae (ed. T. Wiederholm). - Entomologica Scandinavica Supplements 19: 33–112.

19. Cranston P.S., Oliver D.R. & Saether O.A. 1983: The larvae of Orthocladiinae (Diptera: Chironomidae) of the Holarctic region - Keys and diagnosis. In: Chironomidae of the Holarctic region - Keys and diagnosis. Part 1. Larvae (ed. T. Wiederholm). - Entomologica Scandinavica Supplement 19: 149–291.

20. Pinder L.C.V. & Reiss F. 1983: 10. The larvae of Chironominae (Diptera: Chironomidae) of the Holarctic region - Keys and diagnosis. In: Chironomidae of the Holarctic region - Keys and diagnosis. Part 1. Larvae (ed. T. Wiederholm). - Entomologica Scandinavica Supplement 19: 293–436.

21. Antczak-Orlewska O., Płóciennik M., Sobczyk R., Okupny D., Stachowicz-Rybka R., Rzodkiewicz M., Sicinski J., Mroczkowska A., Krapiec M., Słowinski M. & Kittel P. 2021: Chironomidae Morphological Types and Functional Feeding Groups as a Habitat Complexity Vestige. - Frontiers in Ecology and Evolution 8: 583831.

22. Hirvenoja M. 1973: Revision der Gattung Cricotopus van der Wulp und ihrer Verwandten (Diptera, Chironomidae). – Annales Zoologici Fennici 10: 1–363.

23. Moller-Pillot H.K.M. 1984: De larven der Nederlandse Chironomidae (Diptera). Orthocladiinae sensu lato. - Nederlandse Faunistische Mededelingen 1B: 1–176.

24. Heino J. 2008: Patterns of functional biodiversity and function–environment relationships in lake littoral macroinvertebrates. - Limnol. Oceanogr. 53: 1446–1455.

25. Berg, M. B.-1995. Larval food and feeding behavior, in The Chironomidae: Biology and Ecology of Non-biting Chironomids, eds P. D. Armitage, P. S. Cranston, and L. V. Pinder (London: Chapman and Hall), 136–198. DOI: 10. 1007/978–94–011-0715-0_7.

26. Oliver 1971: Life story of Chironomidae. - Annual Review of Entomology 16: 211–230.

27. Mackey A.P. 1979: Trophic dependencies of some larval Chironomidae (Diptera) and fish species in the River Thames. - Hydrobiologia 62: 241–247.

28. Loden M.S. 1974: Predation by chironomid (Diptera) larvae on oligochaetes. - Limnology and Oceanography 19: 156–159.
