# Supplementary material for: Wetland productivity determines trade‐off between biodiversity support and greenhouse gas production
Source: Ecol Evol. 2023 Oct 20;13(10):e10619. doi: 10.1002/ece3.10619 (PMC10587742; doi:10.1002/ece3.10619)
Supplement: Supplementary file 22 — Data S1 [file ECE3-13-e10619-s021.docx]

Appendix table 1: Site number, latitudinal- and longitudinal coordinates [WGS-84], total surface area [ha], and category.

Appendix table 2: Genus abundance in feeding groups. If divisions were not integers, abundance was rounded down. Genera lacking affiliation deemed “Undetermined”. If only one individual for a multi affiliation genus, one was added to each feeding group. Citations to feeding group determination in superscript (in red: inference by Brodin,Y (2022) based on larval mouthparts).

Appendix table 3: Means and ranges of raw data used for linear mixed-effects models, divided between sampled locations.

Appendix table 4: Structure of final linear mixed-effects models where location was set as random effect. Any variable represented with a response in the table, refers to that variable being included in the final model, Significance follow Type II Walds χ^2^ response (P>0.10 = NS, 0.05<P<0.10 = (´), 0.01<P<0.05 = (*), 0.001<P<0.01 = (**), P<0.001 = (***)).

Appendix figure 1: Aerial photograph of site 1

Appendix figure 2: Aerial photograph of site 2

Appendix figure 3: Aerial photograph of site 3

Appendix figure 4: Aerial photograph of site 4

Appendix figure 5: Aerial photograph of site 5

Appendix figure 6: Aerial photograph of site 6

Appendix figure 7: Aerial photograph of site 7

Appendix figure 8: Aerial photograph of site 8

Appendix figure 9: Aerial photograph of site 9

Appendix figure 10: Aerial photograph of site 10

Appendix figure 11: Aerial photograph of site 11

Appendix figure 12: Aerial photograph of site 12

Appendix figure 13: Aerial photograph of site 13

Appendix figure 14: Aerial photograph of site 14

Appendix figure 15: Aerial photograph of site 15

Appendix figure 16: Aerial photograph of site 16

Appendix figure 17: Aerial photograph of site 17
